# Responses of the Housefly, *Musca domestica*, to the Hytrosavirus Replication: Impacts on Host's Vitellogenesis and Immunity

**DOI:** 10.3389/fmicb.2017.00583

**Published:** 2017-04-05

**Authors:** Henry M. Kariithi, Xu Yao, Fahong Yu, Peter E. Teal, Chelsea P. Verhoeven, Drion G. Boucias

**Affiliations:** ^1^Biotechnology Research Institute, Kenya Agricultural and Livestock Research OrganizationNairobi, Kenya; ^2^Insect Pest Control Laboratory, Joint FAO/IAEA Division of Nuclear Techniques in Food and AgricultureVienna, Austria; ^3^Entomology and Nematology Department, University of FloridaGainesville, FL, USA; ^4^Interdisciplinary Centre for Biotechnology Research, University of FloridaGainesville, FL, USA; ^5^Center for Medical, Agricultural and Veterinary Entomology, USDA, ARSGainesville, FL, USA

**Keywords:** *Hytrosaviridae*, hypertrophy, sesquiterpenoids, vitellogenesis, corpora allata/corpora cardiaca complex, immunity pathways

## Abstract

*Hytrosaviridae* family members replicate in the salivary glands (SGs) of their adult dipteran hosts and are transmitted to uninfected hosts via saliva during feeding. Despite inducing similar gross symptoms (SG hypertrophy; SGH), hytrosaviruses (SGHVs) have distinct pathobiologies, including sex-ratio distortions in tsetse flies and refusal of infected housefly females to copulate. Via unknown mechanism(s), SGHV replication in other tissues results in reduced fecundity in tsetse flies and total shutdown of vitellogenesis and sterility in housefly females. We hypothesized that vitellogenesis shutdown was caused by virus-induced modulation of hormonal titers. Here, we used RNA-Seq to investigate virus-induced modulation of host genes/pathways in healthy and virus-infected houseflies, and we validated expression of modulated genes (*n* = 23) by RT-qPCR. We also evaluated the levels and activities of hemolymph AMPs, levels of endogenous sesquiterpenoids, and impacts of exogenous hormones on ovarian development in viremic females. Of the 973 housefly unigenes that were significantly modulated (padj ≤ 0.01, log2FC ≤ −2.0 or ≥ 2.0), 446 and 527 genes were downregulated and upregulated, respectively. While the most downregulated genes were related to reproduction (embryogenesis/oogenesis), the repertoire of upregulated genes was overrepresented by genes related to non-self recognition, ubiquitin-protease system, cytoskeletal traffic, cellular proliferation, development and movement, and snRNA processing. Overall, the virus, *Musca domestica* salivary gland hytrosavirus (MdSGHV), induced the upregulation of various components of the siRNA, innate antimicrobial immune, and autophagy pathways. We show that MdSGHV undergo limited morphogenesis in the corpora allata/corpora cardiaca (CA/CC) complex of *M. domestica*. MdSGHV replication in CA/CC potentially explains the significant reduction of hemolymph sesquiterpenoids levels, the refusal to mate, and the complete shutdown of egg development in viremic females. Notably, hormonal rescue of vitellogenesis did not result in egg production. The mechanism underlying MdSGHV-induced sterility has yet to be resolved.

## Introduction

The family *Hytrosaviridae* includes a small group of enveloped, rod-shaped dsDNA viruses that infect adult dipterans (Abd-Alla et al., 2009). Hytrosaviruses (SGHVs) are defined by their ability to infect and replicate in the salivary glands (SGs) of their hosts and to induce enlarged, swollen glands within which viral progenies are produced and released into the SG lumen. To date, the adult stage of the blood-feeding tsetse fly (*Glossina* spp.), the filth-feeding housefly (*Musca domestica*), and a phytophagous syrphid *Merodon equestris* have been reported to be SGHV hosts (Abd-Alla et al., 2009). However, the lack of overt symptoms, the endemic prevalence of the infection, the lack of susceptible cell lines, and the chronic nature of SGHV infections have hindered the identification of additional SGHV–fly associations (Kariithi et al., 2013). Within the *Hytrosaviridae* family, GpSGHV infecting *Glossina pallidipes* and MdSGHV infecting *Musca domestica* have been sequenced and characterized (Lietze et al., 2011a). Despite inducing similar gross symptoms, these two viruses possess distinct molecular and pathological properties.

The larger GpSGHV, persisting asymptomatically in tsetse, will under certain conditions induce a symptomatic infection resulting in overt SG hyperplasia (SGH) symptoms via gland cell proliferation. The GpSGHV, due in part to the adenotrophic viviparity exhibited by tsetse, can be transmitted readily via the milk glands to developing progeny (Boucias et al., 2013b). Two GpSGHV lineages have been sequenced. The Ethiopian isolate (GpSGHV-Eth) is associated with higher SGH prevalence (85%) than the Ugandan isolate (GpSGHV-Uga; SGH prevalence of 10%) (Abd-Alla et al., 2008, 2010, 2011, 2016). The observed differential GpSGHV pathologies in different tsetse colonies have been attributed to genetic differences between the two virus isolates. Compared with GpSGHV-Uga, GpSGHV-Eth contains more ORFs, including 24 novel ORFs not found in GpSGHV-Uga. Further, 21% of GpSGHV-Eth ORFs harbor numerous mutations, whereas 6, 7, and 13% of ORFs are deleted, non-canonical, and inserted, respectively (Abd-Alla et al., 2016). At least 60 of the GpSGHV ORFs encode functional proteins, including homologs to well-characterized baculovirus core genes, helicase 2 (DNA repair and recombination) (Wang et al., 2007), LEFs-4, 5, 8, and 9 (transcription), Ac66 (egress of mature virions), and Ac81 (virus–host interactions at late infection stages) (Miele et al., 2011).

The MdSGHV contains a 126 kbp dsDNA genome encoding for 108 ORFs; the virus induces SGH within 3–4 days post challenge (Garcia-Maruniak et al., 2008). Unlike GpSGHV, MdSGHV causes only symptomatic infections in *M. domestica* and induces SGH by cell hypertrophy but not cell proliferation (Lietze et al., 2011a). With this virus, the SGH is due to massive viral DNA replication and morphogenesis in the SG nuclei, resulting in hypertrophied gland tissue (Lietze et al., 2011a). This virus, although non-lytic, is shed continuously into SG lumens and subsequently deposited in the fly's crop. Adult houseflies exist at high population densities, are gregarious, and feed by regurgitating the contents of the crop (containing saliva–stomach contents) onto the food substrate. Throughout their lifespan, viremic flies release infectious viral particles onto potential food substrates; it is estimated that ~10^6^ viral genome copies are released within a 2–3 s feeding event (Lietze et al., 2009). However, the development of the peritrophic membrane in adult flies serves as an effective barrier to viral ingress in the gut, preventing the development of SGHV-induced epizootics (Prompiboon et al., 2010; Boucias et al., 2015). As an alternative to *per os* acquisition, cuticle wounding has been proposed as an MdSGHV transmission mode (Lietze et al., 2013). As few as 10–100 virus copies delivered into the hemocoel induce the onset of SGH.

Significantly, in houseflies MdSGHV can undergo both DNA replication and transcription in non-SG tissues, but these events do not result in detectable cytopathology (Lietze et al., 2007, 2011b). Using RT-qPCR, Lietze et al. (2007) demonstrated that, whereas MdSGHV transcription significantly occurred in the fat body, tracheal, and brain tissues, negligible transcripts occurred in the midgut, ovaries, and hemolymph. Transcription in the non-SG tissues is believed to negatively impact reproductive fitness of infected females. At the physiological level, MdSGHV infection blocks vitellogenesis, resulting in immediate and permanent female sterilization. The sterility is absolute; to date, none of thousands of SGH-positive females examined have produced eggs. The mechanism(s) underlying the lack of egg production is unknown, but prior qPCR experiments have shown that fat body transcription of selected female-specific yolk proteins is negligible in virus-infected flies (Lietze et al., 2007). In healthy females, the ingestion of a protein meal is believed to trigger a hormonal cascade involving both juvenile hormone (JH) and ecdysone that activates the transcription of yolk proteins in the fat body (Peferoen and de Loof, 1986; Siegenthaler et al., 2010). It should be noted that limited MdSGHV replication has been observed in the corpora cardiaca (CC), the glands responsible for synthesis of sesquiterpenoids. In dipterans, the released JH-III is believed to trigger the follicle epithelium to produce a pulse of ecdysone that activates fat body biosynthesis of egg proteins. Healthy *M. domestica* females, after access to a protein meal, were shown to produce increased levels of hemolymph-borne methyl farnesoate (MF), the unepoxidated form of JH-III (JH-IIIB), a hormone also synthesized in the corpora allata (CA) (Teal et al., 2014). Therefore, MdSGHV infection of the CA–CC complex potentially disrupts the hormonal cascade required for activation of fat body transcription of female-specific proteins. In addition to blocking egg production, viral infection also alters reproductive behavior, most notably, the refusal of infected females to copulate with either healthy or viremic males (Lietze et al., 2007). We speculate that viral transcription in the neural system may suppress vitellogenesis by modulating neuroendrocrine secretion or through direct regulation of fat body biosynthesis.

Recent availability of the *M. domestica* genome (Scott et al., 2014) provides a framework to undertake a global examination of the key genes/pathways being regulated by MdSGHV infection. In this study, RNA-Seq data from triplicate bar-coded libraries were generated on the transcriptomes from healthy and virus-infected (48 h post infection) flies. These data were annotated against the MdSGHV, *M. domestica* and *Drosophila* databases to assess the genes and gene pathways regulated by the SGHV infection. Comparative analysis of the RNA-Seq reads from control and viremic flies demonstrated that MdSGHV infection upregulates components of the innate defense system while suppressing multiple genes involved in oogenesis/egg biosynthesis. Also performed were a series of functional assays targeted at the activities of the antimicrobial peptides (AMPs) and the potential disruption of the hormonal cascade involved in oogenesis in MdSGHV-infected females.

## Materials and methods

### Virus challenge of houseflies

Housefly pupae, obtained from colonies maintained at the USDA Center for Medical, Agricultural and Veterinary Entomology (CMAVE, Gainesville, FL), were placed in rearing cages, provided with deionized water, and reared at 26°C, with a photoperiod of 12 h light and 12 h darkness, and 40% relative humidity until adult emergence. Experiments were conducted with MdSGHV03, the Florida type MdSGHV strain collected in 2005 and subsequently sequenced (Garcia-Maruniak et al., 2008; Prompiboon et al., 2010). Cohorts of synchronously infected houseflies were produced by injecting newly emerged females with filter-sterilized viremic SG homogenates, a treatment that guarantees symptomatic infection in 100% of the injected flies (Lietze et al., 2007). Control flies were injected with sterile phosphate-buffered saline (PBS). Both PBS- and virus-injected flies were maintained in separate groups in the above-described conditions and provided with food and water *ad libitum* until used for sample preparation.

### RNA extraction and quantitation

At 48 h post challenge, PBS- and virus-injected females were placed individually in tubes containing aliquots of 1 ml of Tri-Reagent (Sigma-Aldrich, St. Louis, MO). It should be noted that it is at 48 h-pi that injected flies release numerous infectious viral particles, an indication of active virus replication (Lietze et al., 2009). Each sample was homogenized by adding ~20 zirconium beads (BioSpec Products, Bartlesville, OK) followed by 30 s of vigorous shaking in a bead-homogenizer (FastPrep® Instrument, Qbiogene, Carlsbad, CA). Total RNA was extracted according to the Tri-Reagent protocol. Ethanol-precipitated RNA pellets were suspended in 100 μl DEPC-treated water and treated with RNase-free DNase (Qiagen, Valencia, CA). The isopropanol-precipitated total RNA was re-extracted using SV Total RNA Isolation Kit (Promega, Fitchburg, WI). RNA quantity and quality were assessed using a NanoDrop 2000 spectrophotometer (Thermo Scientific, Wilmington, DE) and the Agilent 2100 Bioanalyzer (Agilent Technologies, Inc., Santa Clara, CA). The absence of contaminating DNA in RNA samples was verified using conventional PCR amplification primers targeting the *28S rRNA* gene (GenBank accession number DQ656974) (Salem et al., 2009).

### RNA-Seq library construction and data analysis

Preparation and sequencing of RNA libraries were performed by ICBR/UF (Gainesville, FL) according to the manufacturer's instructions (Illumina, Inc., San Diego, CA) using the NextSeq500 platform. Briefly, the mRNA was enriched from 1 μg of total RNA per sample using oligo-dT attached to magnetic beads and then subjected to thermal fragmentation using the elute, prime, and fragmentation mix from the Illumina TruSeq™ v2 RNA sample preparation kit. RNA fragments were then converted to double-stranded (ds)-cDNA using reverse transcriptase and random primers provided in the TruSeq RNA sample preparation kit. The ds-cDNA fragments were end-repaired by enzymatic polishing with T4 DNA polymerase and *E. coli* DNA polymerase I Klenow fragment. A single non-templated dA-tail was added to the 3′-end of the repaired fragments and then ligated to NEB adaptors (NEBNext® Ultra RNA library preparation kit). The required fragments were purified by AMPure beads (Agencourt; PN A63881) and enriched by PCR amplification. The amplified libraries were purified and quantified using the Agilent DNA high-sensitivity kit on an Agilent 2100 Bioanalyzer (Agilent Technologies, Inc.) and qPCR. Based on the calculated values, the libraries were pooled in equimolar ratios into one pool and sequenced for 2 × 150 bp reads on the Illumina NextSeq 500 platform. Image analysis and base calling were performed using the Illumina Pipeline, where sequence tags were obtained after purity filtering.

Reads acquired from Illumina were cleaned up with the Cutadapt program to trim off sequencing adaptors, low-quality bases with a quality phred-like score <20, and short reads (<40 bases) (Martin, 2011). The genes or transcripts of *M. domestica* (38,323 sequences) from NCBI were used as reference sequences for RNA-Seq analysis. The cleaned reads of each sample were mapped independently against the reference sequences using the mapper of Bowtie 2 with a maximum of three mismatches for each read (Langmead and Salzberg, 2012). The mapping results were processed with Samtools and scripts developed in-house at ICBR to remove potential PCR duplicates and to select unique mapping reads for gene expression estimation. Digital gene expression was determined by counting the numbers of mapped reads for each individual gene counted, using the scripts developed in-house at ICBR and analyzed by the DEB application (Yao and Yu, 2011).

### Assignments of gene ontology (GO) terms and pathway analyses

All genes with padj ≤ 0.2 were selected for the GO analysis. In these selected genes or transcripts, detailed information on the genes was retrieved from the reference databases of *M. domestica* and/or *Drosophila*. In the GO analysis, the levels of upregulation (fold-change > 0) and downregulation (fold-change <0) were based on the log transformed-fold-change of the RNA-seq results. For pathway analysis annotated unigenes selected at high stringency (padj ≤ 0.01) were divided into two pools: the *downregulated* pool (*n* = 446) and an *upregulated* gene pool (*n* = 527), having log2 fold values of ≤ 2.0 and ≥ 2.0, respectively. The amino acid sequences of the *M. domestica* unigenes were blasted against the non-nr-NCBI by BLASTp (*e*-value 10^−4^) and the GOs enriched with Fisher's exact test (cut-off FDR of <0.05) using Blast2GO v4.0 (Conesa et al., 2005). Detailed information of the genes was retrieved from the reference databases of *M. domestica* and/or *Drosophila*. Pathway analyses were performed using the KEGG Mapper v2.7 (Kanehisa et al., 2004) and Insect Innate Immunity Database (IIID) (Brucker et al., 2012). Analysis of the structural features and functional domains of the MdSGHV ORFs was performed using various databases, including PRED-TMBB (Bagos et al., 2004), Pfam v30.0 (Finn et al., 2008), and the NCBI's conserved domain database (CDD) v3.15 (Marchler-Bauer et al., 2015).

### RT-qPCR validation

RNA samples from PBS- and virus-injected female flies were subjected to a one-step RT-qPCR to quantitate the relative transcript abundance of 23 genes selected from the RNA-Seq data. Primers were designed using Primer3 Plus (Untergasser et al., 2007) to amplify 125–200 bp from *M. domestica* genes predicted from the RNA-Seq reads (Table S1). In addition to host gene targets, primers designed to amplify ORFs 1, 10, and 108 (Lietze et al., 2011b) were used to confirm the infection status of the virus-injected samples. The *M. domestica 28S rRNA* gene served as a reference gene and an internal positive control. Using the iTaq™ Universal SYBR® Green One-Step kit (Bio-Rad, CA), each 20-μl reaction contained 50 ng of DNase-treated total RNA, 5 pmol of each gene-specific forward and reverse primer, and the reaction mix with iScript® reverse transcriptase. The one-step RT-qPCR program was one reverse transcription cycle (50°C for 10 min), one initial denaturation cycle (95°C for 3 min,), then 40 cycles of 95°C for 10 s, 60°C for 30 s and melt curve analysis of 65–95°C at 0.5°C increments (5 s per step). The melting peaks were inspected to confirm the presence of a single-amplification PCR product for each reaction. The relative quantification of target gene expression in the virus-injected samples was analyzed using the 2^−ΔΔ*C*T^ method (Livak and Schmittgen, 2001). The qPCR data were presented as the fold change (FC) in the target gene normalized to the *28S rRNA* gene and relative to the PBS-injected control samples. Specifically, the *C*_T_ values of the target genes were subtracted by the *C*_T_ values of *28S rRNA* gene. Then, the FC in the target genes, relative to the PBS-injected control, was calculated for each virus-injected sample and then log_2_-transformed. The log_2_-transformed FC means and standard errors (SE) were determined from the triplicate samples for each target gene.

### Evaluation of endogenous levels of sesquiterpenoids

A series of bioassays were conducted to compare the endogenous levels of JH-III (methyl (2*E*,6*E*)-10,11-epoxy-3,7,11-trimethyl-2,6-dodecadienoate), a JH-IIIB analog (methyl (2*E*,6*E*)-6,7;10,11-bisepoxy-3,7,11-trimethyl-2-dodecenoate), and the JH-III biosynthetic precursor MF (methyl (2*E*,6*E*)-3,7,11-trimethyldodeca-2,6,10-tri-enoate) in MdSGHV-challenged and control flies. Fifty newly emerged females were injected with either sterile saline (control) or with viremic SG homogenates. Flies, provisioned with adult food and water, were incubated at 26°C. At 1, 2, 3, and 4 days post injection (d-pi), flies were cold-immobilized and hemolymph collected by cutting a hind leg and withdrawing extruded hemolymph into a pre-chilled 10 μl capillary. Collected hemolymph samples were transferred into HPLC-grade methanol. The sesquiterpenes in methanol were extracted with pentane to remove lipids and analyzed using a combination of GC and ionization mass-spectroscopy (Teal et al., 2014). Flies used in these extractions were dissected to assess both their SGH status and ovarian development stage.

### Evaluation of impacts of exogenous hormones on MdSGHV-induced pathologies

To evaluate effects of exogenous hormones on ovarian development, females were PBS- and MdSGHV-injected. After 24 h post injection (h-pi), the flies were cold-immobilized and injected (500 ng per fly) with either ecdysone suspended in 10% ethanol, commercial JH-III (Sigma Chemical, St. Louis, MO), or methyl farnesoate (Echelon BioSciences, Salt Lake City, UT) dissolved in acetone (100 mg/ml) and suspended in peanut oil (1 mg/ml). Controls included groups of flies injected with an acetone–peanut oil mixture. The treated females were placed in holding cages with 20 healthy males and provisioned adult food and water. After 5 d-pi, the females were dissected to determine SGH symptoms and ovarian development stages. Another treatment involved injecting a cocktail containing JH-III, MF, and ecdysone mixed with PBS or with SGHV into newly emerged females. Flies were injected with 1 μl containing 500 ng of each hormone suspended in PBS with and without MdSGHV. After 5 d-pi, females were dissected to determine SGH and ovarian development.

Additional groups of females injected initially with either PBS or virus homogenate were incubated for 24 h at 28°C and re-injected with solvent (10% ethanol), 0.1 μg ecdysone, or 1 μg ecdysone. Treated flies were maintained in separate cages on their respective diets of either 10% sucrose in water or water plus adult food (powdered milk + sucrose). After a 2-day incubation period, three females per treatment were collected individually, weighed, and dissected in sterile saline to record SGH and ovarian development. At 4 d-pi, total RNA and cDNA were prepared as described in sections 2.2 and as described in Lietze et al. (2007), followed by RT-qPCR using the iQTM SYBR® Green Supermix (Bio-Rad, Hercules, CA). RT-qPCR was performed using an optimized protocol for three designed primer sets specific for *M. domestica* hexamerin (*Hex2*), yolk protein (*Yo2*), and the *28S rRNA* housekeeping genes (Lietze et al., 2007).

### Impact of MdSGHV infection on the innate defense response

To determine if the increased transcript levels impacted the hemolymph AMP titers and/or microbiome associated with viremic flies, cohorts of newly eclosed females were injected with PBS-homogenates of either healthy or hypertrophied SGs. Additional controls included non-injected females and females injected with *E. coli* D31 (10^6^ cells per fly). At 1–10 d-pi, groups of five flies were homogenized in liquid N_2_, resuspended in 400 μl of 4% acetic acid, vortexed, heated (95°C; 5 min), and centrifuged (12,000 rpm; 15 min; 4°C). Supernatants were lyophilized and stored (−70°C) until assayed. The relative AMP activity was assessed using the inhibition zone assay (Hultmark, 1998). In brief, freeze-dried preparations were solubilized in 30 μl sterile PBS and applied to wells (~2.0 μl per well) of LB agar plates pre-inoculated with early exponential growth phase *Serratia marcescens* or *E. coli* D31. In addition to gram-negative bacteria, plates seeded with a preparation of *Micrococcus luteus* (1 mg/ml, Sigma) or with live *Saccharomyces cerevisiae* were used as substrates to measure relative lysozyme and anti-yeast activities. After 24 h at 28°C, all the above-mentioned plates were examined to estimate the area of inhibition zones. The potential impact of the upregulation of the innate defenses by MdSGHV infection on the housefly microbiome was estimated by calculating the total cultivable colony forming units (CFUs). Three cohorts of newly eclosed female and male adult houseflies were PBS- or MdSGHV-injected and maintained on adult food and water. After 48, 72, and 96 h-pi, flies were individually homogenized for 10 s in 1 ml water using Tissuemiser^TM^ (Fisher Scientific). Resultant homogenates were serially diluted in water and decimal dilutions subsequently spotted (2 μl per spot) onto nutrient agar plates. After 24 h at 28°C, plates were examined to estimate the total CFUs contained in individual flies.

## Results

### Viral (MdSGHV) RNA-Seq reads

**Figure 1:**
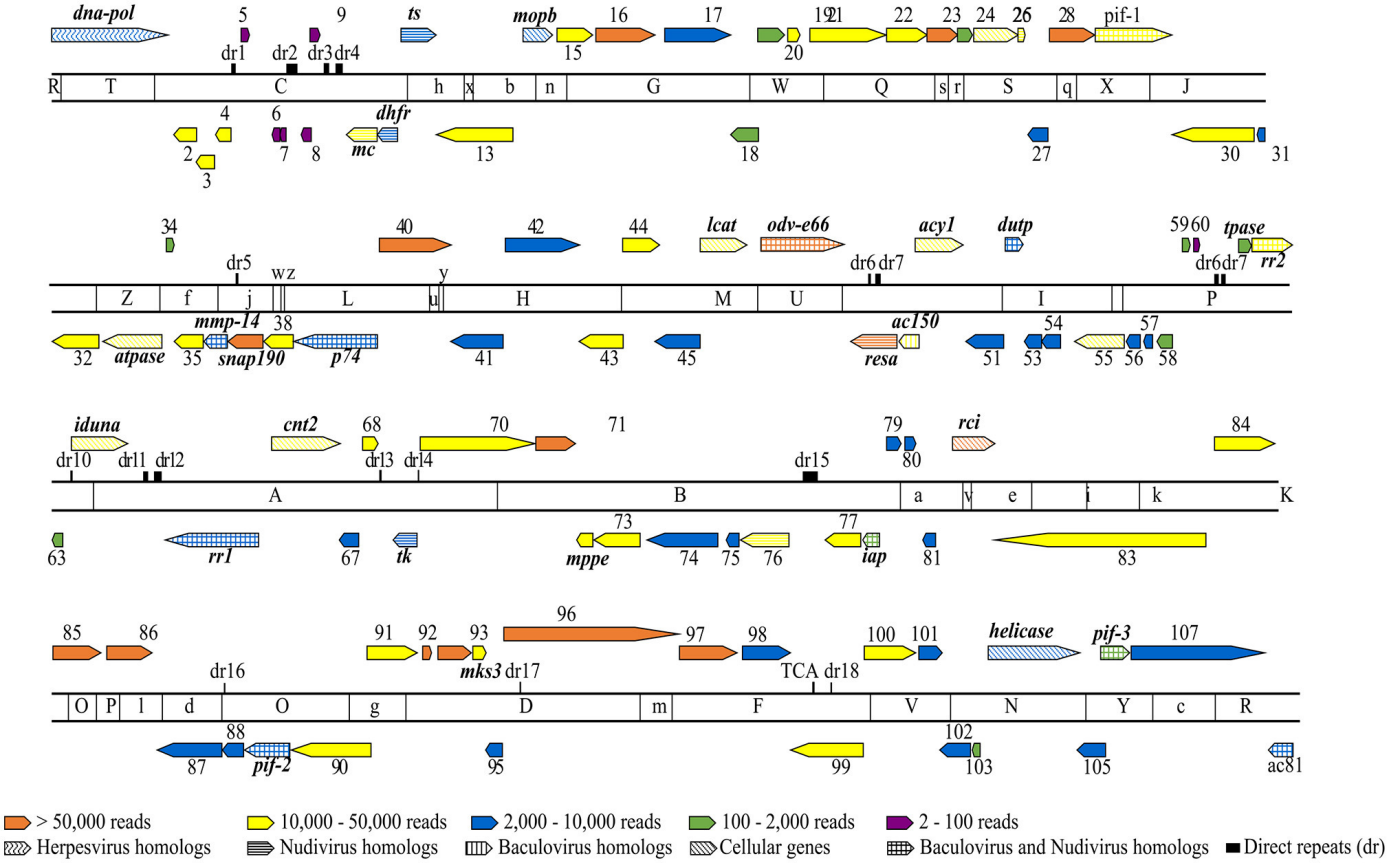
**Overlay of RNA-Seq reads onto a linearized MdSGHV genome and *Eco*RI physical map**. The genome location, relative size and transcriptional direction of each putative ORF is indicated by the arrows. The ORFs are numbered from 1 to 108 with names of the ORFs that could be functionally annotated indicated accordingly. The solid color fills indicate RNA-Seq read counts as follows: 15 ORFs having >50,000 reads (orange), 39 ORFS having 10,000–50,000 reads (yellow), 37 ORFS having 2,000–10,000 reads (blue), 11 ORFs having 100–2,000 reads (green) and six ORFs having 2–100 reads (purple). The pattern fills indicate the homologies of MdSGHV genes to known viral and cellular genes. Note that the colored patterns correspond to the colors assigned to respective RNA-Seq read counts. *ts*, thymidylate synthase; *tk*, tyrosine kinase; *pif*, *per os* infectivity factor; *dna-pol*, DNA polymerase; *mmp-14*, zinc-dependent matrix metalloproteinase 14; *snap190*, small nuclear RNA activating complex, subunit 2, SNAP190 Myb; *resa*, ring-infected erythrocyte surface antigen; *rci*, RCI site-specific recombinase; atpase, vacuolar sorting-associated 4A; *iduna*, E3 ubiquitin ligase RNF146; *mks3*, transmembrane protein meckelin (TMEM67); *mc*, mitochondrial carrier; *mppe*, metallophosphoesterase; *rra1/2*, ribonucleoside-diphosphate reductase subunit M1/2; *cnt2*, Na^+^-dependent nucleoside transporter, co-transporter II; *acy1*, M20 aminoacylase-1; *lcat*, lecithin: cholesterol acyltransferase; *mopb*, molybdopterin oxidoreductase; *dupt*, deoxyuridine 5′-triphosphate nucleotidohydrolase; *iap*, death-associated inhibitor of apoptosis 2; *tpase*, transposase.

**Figure 2:**
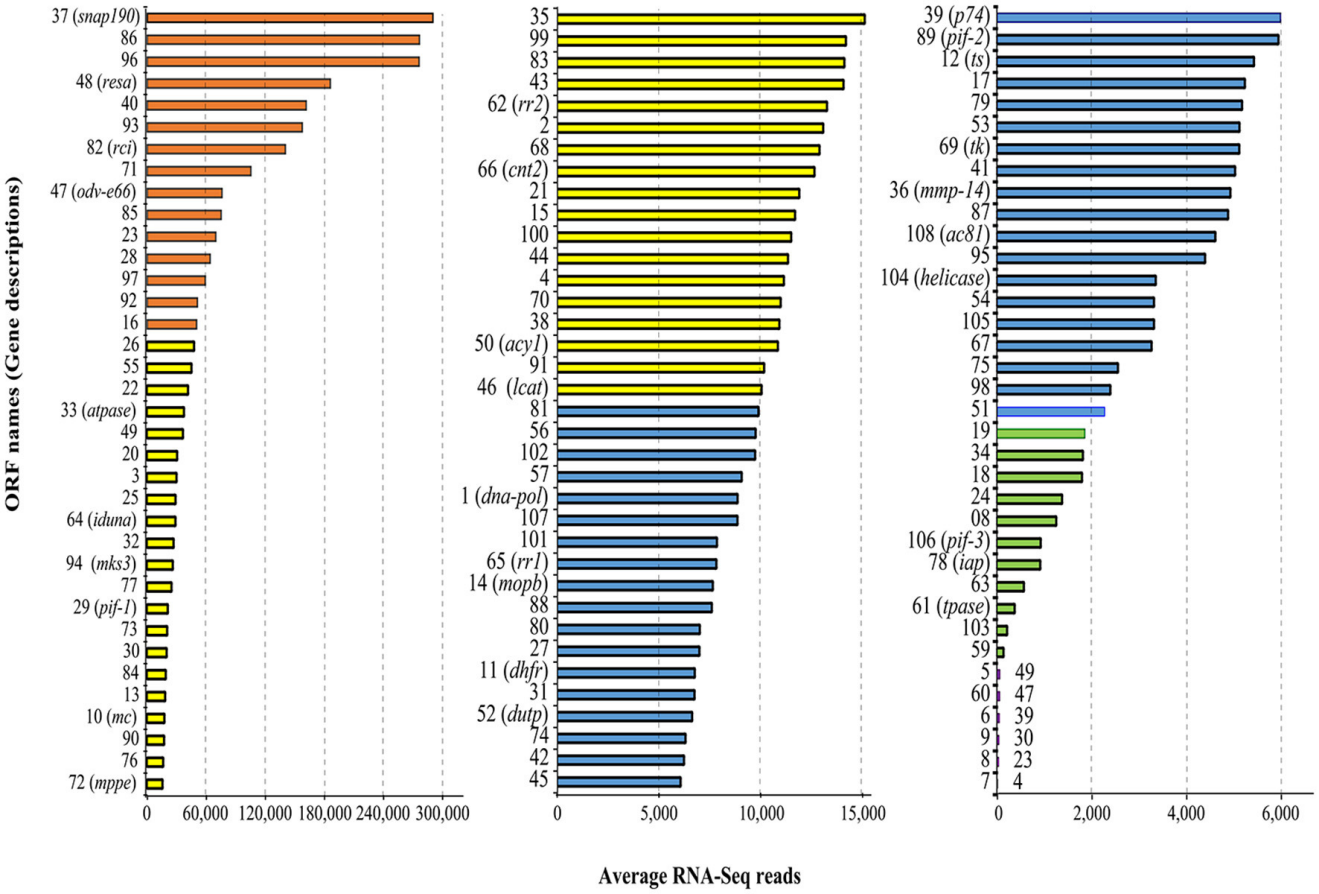
**Average number of RNA-Seq reads in viremic libraries associated with MdSGHV ORFs**. Note the change of scale on x-axis in the three figure panels. The total numbers of the ORFs and the colors in this figure correspond to Figure 1. *ts*, thymidylate synthase; *tk*, tyrosine kinase; *pif*, *per os* infectivity factor; *dna-pol*, DNA polymerase; *mmp-14*, zinc-dependent matrix metalloproteinase 14; *snap190*, small nuclear RNA activating complex, subunit 2, SNAP190 Myb; *resa*, ring-infected erythrocyte surface antigen; *rci*, RCI site-specific recombinase; *atpase*, vacuolar sorting-associated 4A; *iduna*, E3 ubiquitin ligase RNF146; *mks3*, Transmembrane protein Meckelin (TMEM67); *mc*, mitochondrial carrier; *mppe*, metallophosphoesterase; *rra1/2*, ribonucleoside-diphosphate reductase subunit M1/2; *cnt2*, Na^+^-dependent nucleoside transporter, co-transporter II; *acy1*, M20 Aminoacylase-1; *lcat*, lecithin: cholesterol acyltransferase; *mopb*, molybdopterin oxidoreductase; *dupt*, deoxyuridine 5′-triphosphate nucleotidohydrolase; *iap*, death-associated inhibitor of apoptosis 2; *tpase*, transposase.

Mapping the RNA-Seq data onto the MdSGHV genome (GenBank accession number NC_010671) demonstrated that the three PBS-injected (healthy) RNA pools had a total of only 1,528 reads (out of >2.8 × 10^7^ unique mapped reads obtained) that mapped onto the MdSGHV ORFs. In general, the reads detected in the PBS-injected samples mapped mainly onto the highly expressed virus ORFs 37 (a small nuclear RNA-activating complex subunit 2 (SnAPC-2)-like protein), 40, 48 (a *N. meningitidis* TspB virulence factor-like protein), 86 (matrix protein), MdSGHV093, and MdSGHV096 (Table S2). Alternatively, at 48 h-pi, the RNA-Seq data from the three virus-infected RNA pools contained 4.5 × 10^6^, 4.2 × 10^6^, and 2.0 × 10^6^ reads, respectively, that could be mapped onto all of the 108 MdSGHV ORFs (Garcia-Maruniak et al., 2009) (Figure 1). Overall, the number of reads was not correlated (*R*^2^ = 0.056) to size of the ORFs; however, the six ORFs having the smallest number of reads were >300 bp in length (Figure S1). Based on the read frequencies, viral transcription appeared to be regulated; 15 ORFs (highly abundant) had more than 5.0 × 10^4^ reads per library, 39 ORFs (abundant) had between 1.0 and 5.0 × 10^4^ reads per library, 37 ORFs (moderate) had between 10.0 and 2.0 × 10^3^ reads per library, and 11 ORFs (low) had between 20.0 and 2.0 × 10^2^ reads per library (Figure 2).

### Host (*M. domestica*) RNA-Seq reads

The three libraries generated for the individual PBS-control and MdSGHV-infected females produced similar numbers of raw forward and reverse reads (Table 1) that mapped onto the *M. domestica* genome (GenBank accession number AQPM00000000.1). The reduced number of unique mapped reads detected in virus-infected libraries is likely due to the presence of the millions of reads matching the viral ORFs that were absent in healthy *M. domestica* (see prior section). After filtering out low-quality mappings, 20,197 genes were selected for further analysis. In the RNA-Seq analyses, genes with low (<10 reads) or no reads in any of the libraries were discarded, leaving ~17,000 putative gene targets. Comparative analysis between the mapped reads (17,000 putative genes) of the healthy (control) and infected libraries identified ~2,300 (14%) and ~5,500 (32%) having padj ≤ 0.01 and <0.2, respectively, that were modulated to various levels by MdSGHV infection. Of the ~5,500 differentially expressed genes (padj <0.2), similar numbers were placed into the positive log2FC (2,7) and the negative log2FC (−2,7) pools. Within these unigene gene pools (padj <0.2), 858 and 559 unigenes had log2FC of ≥2.0 or ≤ −2.0, respectively.

**Table 1:** **Summary statistics of the NextSeq FC bidirectional reads (R1 and R2) generated on the six libraries prepared from total RNA extracted from the PBS-injected (Control) and virus-injected (MdSGHV) *Musca domestica* female flies**.

**RNA sample**	**Raw data**		**Cleanup data**		**mRNA mapped reads^a^**		**mRNA unique mapped reads^a^**
	**R1**	**R2**	**R1**	**R2**	**R1**	**R2**	**R1 + R2**
Control 3	4.91E+07	4.91E+07	4.91E+07	4.91E+07	4.09E+07	3.81E+07	1.05E+07
Control 4	4.83E+07	4.83E+07	4.83E+07	4.83E+07	3.91E+07	3.70E+07	9.96E+06
Control 5	3.86E+07	3.86E+07	3.86E+07	3.86E+07	2.93E+07	2.77E+07	7.45E+06
MdSGHV 2	5.26E+07	5.26E+07	5.26E+07	5.26E+07	2.84E+07	2.71E+07	7.69E+06
MdSGHV 4	5.01E+07	5.01E+07	5.00E+07	5.01E+07	3.10E+07	2.95E+07	8.61E+06
MdSGHV 5	5.05E+07	5.05E+07	5.04E+07	5.05E+07	2.99E+07	2.85E+07	8.29E+06

a *Includes reads that could be mapped against the reference sequences of the housefly (M. domestica). The additional reads found in the MdSGHV libraries could be mapped to the ORFs encoded by the MdSGHV genome*.

### Validation of RNA-Seq data by RT-qPCR

**Figure 3:**
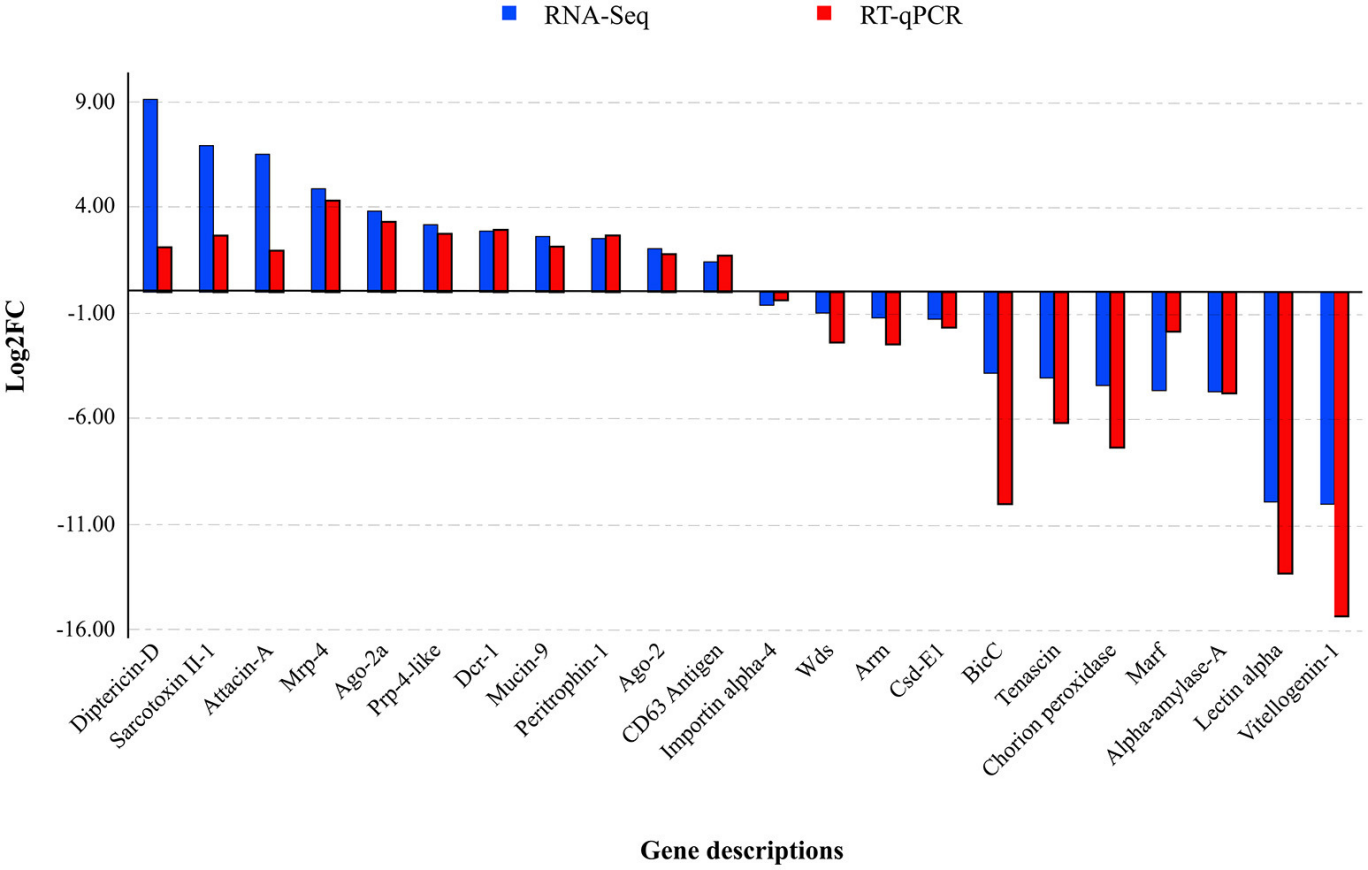
**MdSGHV-induced modulation of expression of 22 host genes**. Except few disparities such as pronounced log2FC of diptericin-D, sarcotoxin II-1, and attacin-A, the RT-qPCR quantification of the host genes correlated with the RNA-Seq data. *Mrp-4*, Multi-drug resistance-associated protein-4; *Prp-4-like*, Proline-rich protein 4-like; *Wds*, Protein will die slowly; *Csd-E1*, Cold shock domain-containing protein-E1; *BicC*, Protein bicaudal C; *Marf*, Meiosis arrest female protein; *Argo*, Argonaute, *Dcr*, Endoribonuclease dicer.

The RT-qPCR assays targeting MdSGHV ORFs 1 (DNA pol), 10 (mitochondrial carrier), and 108 (Ac81) demonstrated that the control libraries lacked the virus (*C*_T_ values >35). Further, the copy numbers of MdSGHV010, a gene having an abundant level of mapped reads in the RNA-Seq analysis, was estimated to be seven-fold greater than those generated for the moderately expressed MdSGHV001 (Figure 2). The qPCR assays, conducted on 22 host genes regulated to varying degrees by MdSGHV infection, produced data that supported the RNA-Seq findings (Figure 3). In certain cases, there were disparities in the fold-difference between these data sets. For example, the pronounced increase (128- to 512-fold) in the transcript levels for cationic peptides (diptericin-D, sarcotoxin II-1, and attacin-A) observed in the RNA-Seq data were not observed in the qPCR assays; in these reactions, only 4- to 8-fold increases were recorded for these gene targets. On the other hand, RT-qPCR data recorded lower transcript levels in eight of the eleven genes identified by RNA-Seq analysis to be suppressed by MdSGHV infection (Figure 3). It should be noted that the variation between RNA-Seq and RT-qPCR data may be due to biological replication; the qPCR analyses were conducted on RNA preparations that were not the same as the RNA preparations used to prepare the libraries for the RNA-Seq analyses.

### Gene ontology (GO) classification

**Figure 4:**
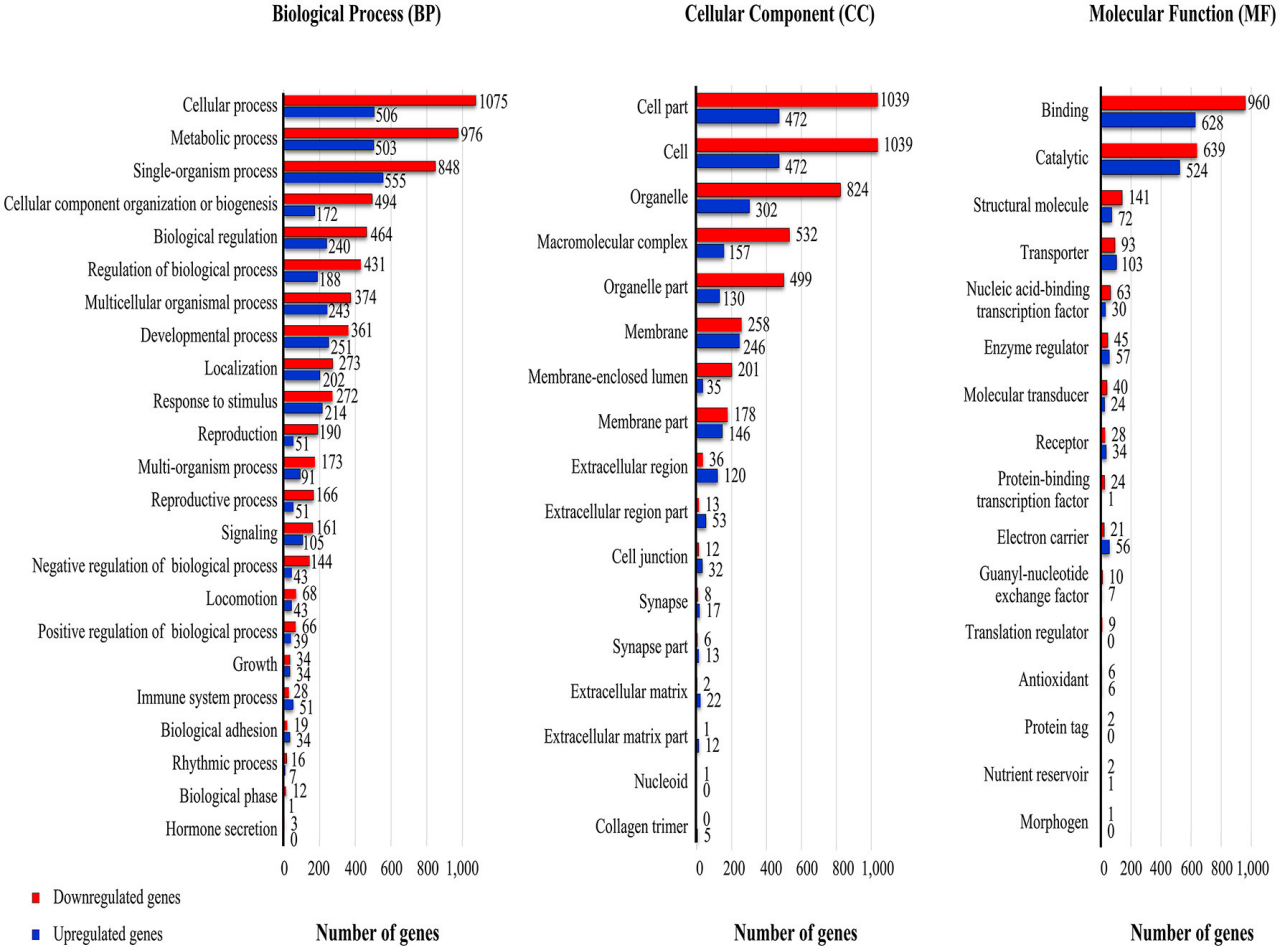
**GO terms associated with RNA-Seq reads from viremic and healthy housefly libraries**. The GO terms were mapped to individual genes that had a padj <= 0.2. The levels of upregulation (red, fold change > 2.0) and downregulation (blue, fold change < −2.0) were based on the log transformed-fold-change of the RNA-Seq reads.

Many of the differentially expressed host unigenes (padj <0.2) could be annotated and assigned via GO annotation into the three major categories: the biological process (BP; 2,271 unigenes), the cellular component (CP; 1,980 unigenes), and the molecular function (MF; 2,462 unigenes) ontologies (Figure 4). Within the major GO categories, genes having negative log2FC values (*downregulated* category) outnumbered by ~1.5- to 2-fold those genes having positive log2FC values (*upregulated* category). It should be noted that increased levels within the *downregulated* category in the GO analysis is somewhat biased; more unigenes (1,664) that could not be assigned a GO category (*n* = 2,713) had a positive log2FC value.

Viral infection at 48 h-pi altered transcription profiles in many of the GO subcategories within the BP, CP, and MF ontology (Figure 4). Within most of the BP subcategories, there were 1.3- to 2.6-fold more genes having *downregulated* transcript levels compared to the controls. The three subcategories of reproduction, reproductive process, and negative regulation of biological process had more than a 3-fold increase in the number of *downregulated* transcripts. There were exceptions to the general reduction in transcript levels; for example, both immune and biological adhesion subcategories within the BP ontology contained more genes having a positive than a negative log2FC. Like the BP, within the CP, the majority of subcategories had reduced transcript levels; marked *downregulation* was noted with transcripts in the membrane-enclosed lumen extracellular region category. This subcategory included genes associated with organelles enclosed by double membranes (endoplasmic reticulum, nucleus). However, the extracellular space and matrix and cell junction subcategories contained more genes with *upregulated* than *downregulated* transcript levels. Within the MF, the overall degree of the gene *downregulation* induced by MdSGHV infection was somewhat less than in the BP and CP categories. In the MF category, the transporter activity, enzyme regulatory, electron carrier, and receptor subcategories had more unigenes having a positive than a negative log2FC.

### Differentially expressed host genes

Two sets of unigenes (padj ≤ 0.01) exhibiting the greatest log2FC reductions or increases in transcript levels were annotated against a combination of *M. domestica* and *Drosophila* databases. Annotated genes having the lowest log2FC values (2^−10^ to 2^−4^) were dominated by the genes encoding for female-specific proteins related to reproduction, egg production, and embryogenesis (yolk proteins, vitellogenins, nudel, hexamerin, meiosis arrest female 1, protein takeout, bicaudal C, chorion peroxidase, pendulin) (Table S3). In addition, other annotated genes expressing high log2FC reductions included transcripts encoding for proteins involved in non-self-recognition (lectin subunit alpha); SG associated endonucleases (Tsal1 precursor, Tsal2-A); ubiquitin-proteasome (E3 ubiquitin-ligase UBR1, F-box only 33, MKRN2 opposite strand protein); intracellular movement (kinesin-like protein); lipid metabolism (phospholipid-transporting ATPase IF, acyl-COA binding–like, mitochondrial D-beta-hydroxybutyrate); starch metabolism (amylases); cell proliferation, differentiation, and movement (dual specificity tyrosine-phosphorylation-regulated kinase 3 homolog, epithelial discoidin domain containing receptor-1 serine/threonine aurora 2); and snRNA processing (integrator complex-10).

The 50 genes having the greatest log2FC increases (2^4^ to 2^9^) included 45 annotated genes, nearly half of which were components or products of humoral and cellular innate defense responses (Table S4). These included various cationic peptides (attacins, sarcotoxins/cecropins, and diptericins), apoptosis-related genes (peroxiredoxin 3), transcriptional factors (relish), coagulation components (proclotting enzyme), and endocytosis (low density liporeceptor adapter 1, acyl-COA-thioesterase 1, and calpain-C). Other genes with increased transcript levels encoded for extracellular matrix-associated proteins (laminin subunit gamma-1, heparan sulfate 2-O-sulfotransferase pipe, prolyl 4-hydroxylase-α-2, fibronectin type III domain-containing 5 – isoform, dachsous), glycosyl hydrolases (maltases, α-*N*-acetylgalactosaminidase), stress-associated (Hsp70), lipid metabolism (elongation of very long-chain fatty acid protein, low-density lipoprotein receptor adapter protein 1-A), transposable element (blastopia polyprotein), cuticle synthesis (cuticle 8, pupal cuticle G1A, laccase, larval cuticle-8), and RNA-binding proteins (couch potato).

### Pathway analysis

In total, 124 of the 973 annotated unigenes (padj ≤ 0.01, log2FC ≤ −2.0 or ≥ 2.0) could be placed into one or more of 101 KEGG pathways. The low number of genes that could be assigned accurately to specific target pathways is probably due to the nature of multifunctional proteins, which perform several functions simultaneously or not. Only 42 of the 446 *downregulated* genes could be placed into one or more of 56 KEGG pathways (Table S5); 25 pathways contained only *downregulated* (≤ −2.0) genes. Within this pool of 25 pathways, MdSGHV infection lowered the transcript levels of various genes involved in nucleic acid replication and transport (α-primase complex, MCM complex, helicase, and nucleoporins Ndc1 and Nup153) and in DNA repair pathways (endonucleases, DNA excision repair protein, DNA polymerases, and exonuclease). Additionally, components of the Wnt signaling pathways, including the F-box/WD repeat-containing protein 11, serine/threonine-protein (nemo-like) kinase NLK, vang-like protein 2-B, and 1-phosphatidylinositol 4,5-bisphosphate phosphodiesterase, had reduced transcript levels in infected females. These transcripts were distributed within branches of the canonical pathway, the planar cell polarity (PCP) pathway, and the Wnt/Ca^2+^-pathway.

In total, 82 of the 527 *upregulated* genes could be placed into one or more of 70 KEGG pathways (Table S6); 39 pathways contained only *upregulated* transcripts. Within the 39 *upregulated* pathways pool, increased transcript levels of enzymes (UDP-glucuronosyltransferases, uridine phosphorylase, glutathione S-transferases, dihydropyrimidine dehydrogenases, and xanthine dehydrogenase) involved in xenobiotic (drug) biodegradation and metabolism were induced by MdSGHV infection. Viremic flies had increased transcript titers of genes involved in carbohydrate metabolism, including those associated with chitin, glycan, amino-sugar, and pentose metabolism.

The 31 pathways containing a mix of *upregulated* and *downregulated* transcripts demonstrated that the differentially expressed transcripts often targeted different sections/branches of a pathway (see Tables S5, S6). For example, in the pyrimidine and purine pathways, the *downregulated* transcripts encoded for enzymes involved in nucleic acid strand synthesis, whereas the *upregulated* transcripts encoded enzymes targeting nucleotide catalysis/turnover (5′ nucleotidase, adenosine deaminase, adenylate cyclase). The insect hormone pathway contained reduced transcript levels of cytochrome P450 307a1 (*spook*) and cytochrome P450 18a1 involved in ecdysone biosynthesis and 20-hydroxyecdysone (20-E) inactivation, respectively, and increased transcript levels of enzymes involved in MF and JH-III biosynthesis. In the extracellular matrix (ECM) pathway, the collagen transcript level was increased, whereas the laminin transcript level was reduced. Certain pathways contained a combination of multifunctional genes and pathway-specific genes identified either in the *upregulated* or *downregulated* transcript pools. For example, the hippo signaling pathway contained several multifunctional *upregulated* genes (protocadherin and F-actin) and various *downregulated* genes that include pathway-specific serine/threonine-protein kinase Warts.

Due in part to the inability to map the annotated innate defense genes into KEGG pathways, a Top BLASTp search on the IIID database was conducted on the 973 annotated unigenes (padj ≤ 0.01, log2FC ≤ −2.0 or ≥ 2.0). Forty-two out of the 527 *upregulated* genes were identified as components/products of both the humoral and cellular immune pathways (Table 2). These included genes encoding for the cationic peptides (AMPs), components of the Toll and Imd pathways, nonself-recognition proteins, phenoloxidase cascade, antiviral defense, and stress-related proteins. Only 12 of the 446 *downregulated* genes could be associated with genes in the IIID (Table 3). In most cases, these associations reflected the dual role that components of some of these pathways may play in insect immunity, reproduction, and development. For example, the female-specific hexamerin and vitellogenin receptor are associated with vitellogenesis and chorion peroxidase, nudel and nanos are involved in embryogenesis. In this list, there were genes associated with the negative regulation of Jak/STAT, including E3 SUMO-protein ligase PIAS2 that, when activated by phosphorylation (aurora kinase), inhibits STAT signaling.

**Table 2:** **Top BLASTp scores of immunity-related proteins upregulated in the MdSGHV-infected *Musca domestica* flies**.

**Description of** ***Musca domestica*** **proteins**			**Description of sequences producing significant alignments**					**Pathway**	**Processes and roles in insect immunity**
**Accession No**.	**Protein name**	**Log2FC**	**Best match protein name; [species name]**	**Identity [%]**	**Accession No**.	**Bits score**	***E*-value**		
XP_005187576.1	Diptericin-D-like	9.13E+00	Diptericin (dpt B); [*D. melanogaster*]	61	NP_523787.2	89	3e-20	AMP	Antibacterial peptide; Induced by pathogen infection
XP_005179713.1	Cecropin-A2-like	8.16E+00	Cecropin A2; [*D. melanogaster*]	79	NP_524589.1	86.7	1e-19	AMP	Antibacterial/antifungal peptide; Induced by infection
XP_005180079.2	Sarcotoxin-2A-like	6.94E+00	Attacin A (Att A); [*D. melanogaster*]	32	NP_523745.1	110	7e-26	AMP	Antibacterial peptide; is induced by infection
XP_005179714.1	Sarcotoxin-1C-like	6.76E+00	Cecropin A2; [*D. melanogaster*]	76	NP_524589.1	99.8	1e-23	AMP	Antibacterial/antifungal peptide; Induced by infection
XP_005178516.1	Attacin-A-like	6.53E+00	Attacin A (Att A); [*D. melanogaster*]	37	NP_523745.1	139	4e-35	AMP	Antibacterial peptide; is induced by infection
NP_001295990.1	Attacin-A-like precursor	6.18E+00	Attacin A (Att A); [*D. melanogaster*]	43	NP_523745.1	165	1e-42	AMP	Antibacterial peptide; is induced by infection
XP_005178550.1	Attacin-A-like	5.79E+00	Attacin A (Att A); [*D. melanogaster*]	37	NP_523745.1	134	1e-33	AMP	Antibacterial peptide; is induced by infection
XP_005191037.2	Nuclear factor NF-kappa-B p110 subunit-like	5.53E+00	Relish protein; [*D. melanogaster*]	21	AAF20134.1	72	6e-14	IMD	A P105-like transcription factor
XP_005182912.1	Low density lipoprotein receptor adapter protein 1-A-like	5.43E+00	Uncharacterized protein CG4393; [*D. melanogaster*]	27	NP_651143.2	55.5	2e-09	Toll	Ankyrin repeat and sterile alpha motif domain containing 1B protein. A weak homolog of the mammalian TNF receptor-associated factor 3 (TRAF3) gene
XP_005187575.1	Diptericin-D-like	5.15E+00	Diptericin (dpt B); [*D. melanogaster*]	61	NP_523787.2	87.4	7e-20	AMP	Antibacterial peptide; is induced by infection
XP_011291120.1	Tyrosine-protein kinase Fps85D	5.10E+00	Hopscotch; [*A. mellifera*]	36	XP_001121783.1	168	7e-43	JAK/STAT	Cellular pathway
XP_005185225.1	Proclotting enzyme	5.09E+00	Class II-associated invariant chain peptide (CLIPD3); [*An. gambiae*]	70	XP_321698.3	498	1e-142	Toll	Clip or disulphide knot domain protein; The protein is present in horseshoe crab proclotting enzyme N-terminal domain, *Drosophila* Easter & silkworm prophenoloxidase-activating enzyme; Trypsin like serine protease
XP_005175934.1	peroxiredoxin-2-like	5.09E+00	Thioredoxin-1 protein (TPX1); [*An. gambiae*]	69	XP_310704.3	323	3e-90	Humoral Response	Thioredoxin-dependent peroxidase
XP_005191014.2	Heat shock protein 70	4.63E+00	Heat shock protein 70Aa; [*A. pisum*]	81	XP_001951915.1	956	0.0	JAK/STAT	Involved in general stress response
XP_005178152.1	Chymotrypsinogen 2	4.53E+00	Class II-associated invariant chain peptide (CLIPA3); [*An. gambiae*]	54	XP_307499.3	380	1e-107	Toll	Clip or disulphide knot domain protein; The protein is present in horseshoe crab proclotting enzyme N-terminal domain, *Drosophila* Easter & silkworm prophenoloxidase-activating enzyme; Trypsin like serine protease
XP_005187846.1	Cuticle protein 8	4.47E+00	Gram-negative binding protein-like protein-3 (GNBP-like 3) protein; [*D. melanogaster*]	62	NP_647873.2	83.6	2e-18	Toll	Antibacterial defense response; needed to present Gram-positive peptidoglycan (PG) to peptidoglycan recognition protein SA (PGRP-SA)
XP_005177649.2	Laccase-2-like	4.47E+00	Multicopper oxidase-1 (Mco1) protein; [*D. melanogaster*]	35	ADV37005.1	417	1e-118	Humoral Response	A ferroxidase essential for iron homeostasis in *D. melanogaster*
XP_005174948.1	Larval cuticle protein 8-like isoform X1	4.28E+00	Cuticular protein 67Fb; [*D. melanogaster*]	37	NP_648420.1	53.9	1e-09	Unknown Pathway	A structural components of the cuticle (chitin); upregulated during nematode infection in insects
XP_011294503.1	Fibrinogen alpha chain	4.23E+00	Angiopoietin; [*A. mellifera*]	88	XP_395740.2	505	1e-144	AMP	Involved in pathogen recognition acting upstream of the antimicrobial effector pathways
XP_005190998.1	Uncharacterized protein LOC101888556	4.09E+00	Uncharacterized protein CG6183; [*D. melanogaster*]	26	AAM29617.1	117	3e-28	Unknown Pathway	The gene encoded small peptides that are potentially novel immune effectors of the gut response in *Drosophila* (upregulated by gut microbiota)
NP_001274154.1	Peptidoglycan-recognition protein SD-like precursor	4.09E+00	Peptidoglycan recognition protein SD (PGRP-SD); [*D. melanogaster*]	50	NP_648145.1	154	2e-39	Toll	Required for Toll activation (sensing) by a subset of Gram positive bacteria; enhances the binding of GNBP1 to Gram-positive PG
XP_005175890.1	Bypass of stop codon protein 1-like	3.92E+00	Uncharacterized protein CG7778; [*D. melanogaster*]	27	AAF52646.1	40.4	6e-05	Unknown Pathway	Potential member of the Glycosylphosphatidyl inositol (GPI) modified protein family
XP_011296144.1	Cell wall protein DAN4	3.90E+00	Serine protease 33 (cSP33); [*A. mellifera*]	81	XP_393316.3	451	1e-128	Toll	Has putative antimicrobial or detoxification functions in *A. mellifera*
XP_005177073.1	Cytochrome P450 CYP12A2-like	3.90E+00	Probable cytochrome P450 4p3 (Cyp4p3); [*D. melanogaster*]	22	NP_610473	80.5	1e-16	Unknown Pathway	Similar to the 4e-37 human cytochrome P450 4B1 protein
XP_005183875.1	protein argonaute-2-like isoform X1	3.85E+00	Argonaute-2; [*D. melanogaster*]	51	AAO39550.1	753	0.0	AMP	Anti-viral defenses
XP_005191037.2	Nuclear factor NF-kappa-B p110 subunit-like	5.53E+00	Relish; [*D. melanogaster*]	21	AAF20134.1	72	6e-14	IMD	A P105-like transcription factor
XP_011292131.1	Uncharacterized protein LOC101887504	3.68E+00	Class II-associated invariant chain peptide (CLIPA10); [*An. gambiae*]	76	XP_308802.2	497	1e-142	Toll	Trypsin like serine protease
XP_005187786.2	Lectin subunit alpha-like	3.67E+00	C-type lectin (CTL)-maltose-binding A6 protein (CTLMA6) [*Ae. aegypti*]	29	XP_001688638.1	60.8	5e-10	IMD	Involved in bacterial recognition, induction of polyphenol oxidase (Ppo) pathway
XP_005187595.1	Laccase-9-like	3.61E+00	Multicopper oxidase-1 (Mco1) protein; [*D. melanogaster*]	34	ADV37005.1	179	1e-46	Humoral Response	Laccase-like protein involved in melanization
XP_005186121.1	Uncharacterized protein LOC101890499	3.49E+00	C-type lectin (CTL)—galactose binding A1 protein (CTLGA1); [*An. gambiae*]	92	XP_319374.3	421	1e-120	IMD	Bacterial recognition, induction of polyphenol oxidase (Ppo) pathway
XP_005180729.2	Fibrinogen C domain-containing protein 1-like	3.49E+00	Conserved hypothetical protein AGAP004916-PA; [*An. gambiae*]	51	XP_001231036.2	224	4e-72	Unknown Pathway	Protein contains fibrinogen-related domains (FReDs)
XP_005188465.2	Anoctamin-6	3.44E+00	Anoctamin; [*D. melanogaster*]	41	AAN13804	644	0.0	Unknown Pathway	A TMEM16 family Ca^2+−^activated Cl^−^ channel in *D. melanogaster* with roles in host defense
XP_005185676.1	Endocuticle structural protein SgAbd-6-like	3.43E+00	Cuticular protein 67Fb; [*D. melanogaster*]	35	NP_648420.1	42.4	5e-06	Unknown Pathway	A structural components of the cuticle (chitin); upregulated during nematode infection in insects
XP_005180889.1	Peptidoglycan-recognition protein LB	3.42E+00	Peptidoglycan recognition protein-LB (PGRP-LB); [*D. melanogaster*]	73	NP_731576.1	276	3e-76	IMD	Cell cycle regulation; PGRP with amidase activity-*in vivo* RNAi indicates a role of PGRP-LB in the down-regulation of the IMD pathway during local and systemic immune response
NP_001273800.1	Uncharacterized protein LOC101900547 precursor	3.40E+00	Chitinase like protein 3; [*A. pisum*]	33	XP_001942596.1	288	2e-79	Toll	Fungal degradation
XP_005187038.1	Peptidoglycan-recognition protein SA-like	3.39E+00	Peptidoglycan recognition protein-SA (PGRP-SA); [*D. melanogaster*]	65	NP_572727.1	246	5e-67	Toll	A protein required for Toll activation by Gram-positive bacteria in flies
XP_005176229.1	flocculation protein FLO11-like isoform X1	3.35E+00	Galectin protein (GalE1); [*A. pisum*]	25	XP_001943769.1	44.7	1e-05	IMD	A galectin with several roles; Upregulated in response to both bacterial and malaria parasite infection in mosquitoes
XP_005191449.1	Usher syndrome type-1G protein homolog	3.27E+00	Cactus, isoform A; [*D. melanogaster*]	34	NP_723960.1	45.4	4e-06	Toll	An ikB-like protein that functions downstream of the Toll signaling pathway
XP_005188441.1	probable chitinase 2	3.26E+00	Chitinase like protein 3; [*A. pisum*]	41	XP_001942596.1	293	8e-81	Toll	Fungal degradation
XP_005190434.2	sodium-dependent nutrient amino acid transporter 1-like	3.24E+00	Uncharacterized protein CG13795; [*D. melanogaster*]	22	AAF52546.4	65.1	3e-12	Unknown Pathway	Dopamine: sodium symporter activity
XP_005175506.1	Uncharacterized protein LOC101889958	3.22E+00	Uncharacterized protein CG13311; [*D. melanogaster*]	30	NP_648258.1	60.5	5e-11	Unknown Pathway	Reportedly upregulated during alcohol exposure in *D. melanogaster*
XP_011291667.1	Uncharacterized protein LOC101888476	3.20E+00	Immunoglobulin (Ig)/fibronectin-3 (Fn3) protein (IgFn 3-14); [*A. mellifera*]	40	XP_392356.3	354	8e-99	Humoral Response	Belongs to the immunoglobulin superfamily genes

*All the 522 proteins upregulated in the MdSGHV-infected flies were used to interrogate the Insect Innate Immunity Database (IIID) (Brucker et al., 2012). A total of 42 protein sequences produced significant hits*.

**Table 3:** **Top BLASTp scores of immunity-related proteins downregulated in the MdSGHV-infected *Musca domestica* flies**.

**Description of** ***Musca domestica*** **proteins**			**Description of sequences producing significant alignments**					**Pathway**	**Processes and roles in insect immunity**
**Accession No**.	**Protein name**	**Log2FC**	**Best match protein name; [species name]**	**Identity [%]**	**Accession No**.	**Bits score**	***E*-value**		
XP_005189999.1	Haemolymph juvenile hormone binding protein (hJHBP)	−4.52E+00	Uncharacterized protein CG16887; [*D. melanogaster*]	39	AAS64701.1	159	1e-40	Unknown Pathway	hJHBP titers are significantly reduced in response to stress larval
XP_005176907.1	Larval serum protein 2-like	−5.80E+00	Hexamerin 70b; [*A. mellifera*]	31	NP_001011600.1	357	1e-100	Humoral Response	–
XP_011295332.1	Vitellogenin receptor	−5.55E+00	Serine protease homolog 54 (SPH54); [*A. mellifera*]	33	XP_396216.2	132	8e-32	Unknown Pathway	Recognition
XP_005176317.1	Ovarian-specific STK (Lok)	−3.34E+00	Immune response deficiency 5, ird5/IKK; [*A. pisum*]	36	XP_001952382.1	174	5e-45	IMD	Recruitment of dsDNA repair proteins
XP_011292728.1	Chorion peroxidase	−4.42E+00	HPX8; [*An. gambiae*]	43	XP_309592.4	577	1e-166	Humoral Response	Required for ovarian follicle maturation
XP_011295171.1	Serine protease nudel	−6.22E+00	SP20; [*A. mellifera*]	33	XP_623911.2	514	1e-146	Toll	Involved in establishment of embryonic dorsal-ventral pathway
XP_005178031.1	Protein Nanos	−4.02E+00	cSPH39; [*A. mellifera*]	43	NP_001035321.1	82.0	3e-17	Unknown Pathway	Functions in migration of primordial germ cells into the gonad in *Drosophila*
XP_005184214.1	CENPA	−4.09E+00	Myd88; [*A. mellifera*]	43	XP_396644.3	64.7	2e-12	Toll	Required for mitotic progression and chromosome segregation
XP_005176745.2	Centrosomal protein of 135 kDa	−3.47E+00	IKKγ-kenny; [*A. mellifera*]	23	XP_001120619.1	56.6	4e-09	IMD	Involved in centriole biogenesis. Acts as a scaffolding protein during early centriole biogenesis
XP_005184457.1	Aurora kinase B	−2.99E+00	RAC-alpha serine/threonine-protein kinase; [*A. mellifera*]	36	XP_396874.3	146	9e-37	JAK/STAT	Functions in attachment of mitotic spindle to centromere
XP_005190494.1	E3 SUMO-protein ligase PIAS2	−2.58E+00	Protein inhibitor of activated STAT; [*A. mellifera*]	52	XP_623571.2	462	1e-131	JAK/STAT	Inhibitor of STAT
XP_005184865.1	Baculoviral IAP repeat-containing protein 5	−2.35E+00	IAP5; [*An. gambiae*]	58	XP_317026.3	127	1e-31	IMD	A multitasking protein that has dual roles in promoting cell proliferation and preventing apoptosis

*All the 446 proteins downregulated in the MdSGHV-infected flies were used to interrogate the Insect Innate Immunity Database (IIID) (Brucker et al., 2012). Only12 protein sequences produced significant hits*.

### Hemolymph levels of sesquiterpenes in viremic and healthy flies

**Figure 5:**
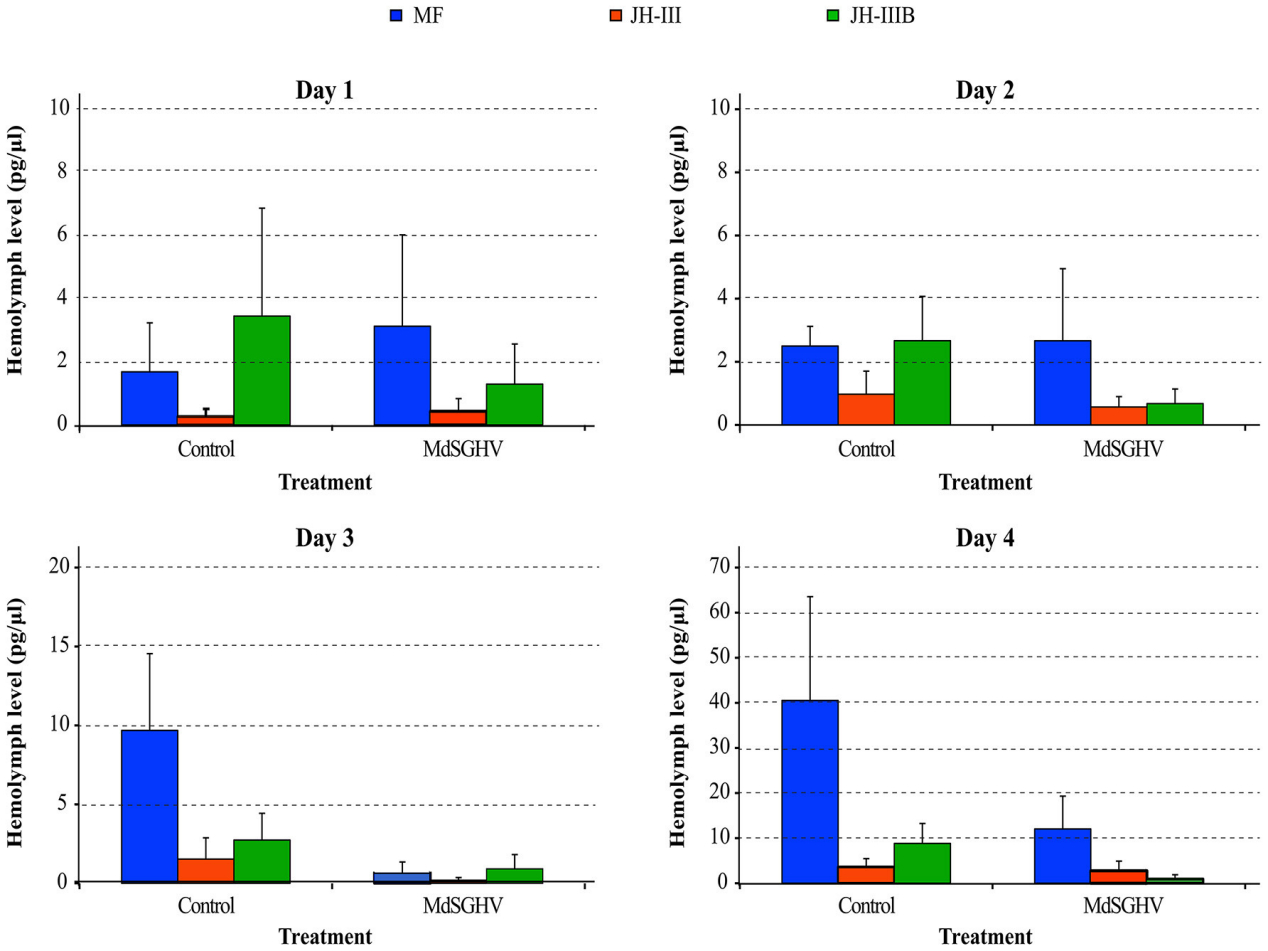
**Levels of sesquiterpenes in viremic and healthy flies over a 4-days experimental period**. Shown are levels of methyl farnesoate (MF; orange), Juvenile hormone-III (JH3; gray) and bisepoxide JH-III (JH3B; green). In agreement with normal ovarian development in healthy females, the MF levels increased > 20-fold during the 4-day experimental period. In viremic flies, MF levels dropped to 4–10-fold less compared to healthy flies. Levels of JH-III and JH-IIIB were lower in viremic flies compared to their healthy counterparts.

Chemical analysis of the hemolymph extracts from PBS- and MdSGHV-injected females revealed fluctuations in titers of MF, JH-III, and JH-IIIB over the 4-day sampling period. In all healthy females, ovarian development reached either stage 3 (advanced vitellogenesis) or stage 4 (fully developed eggs) at 4 days. In these flies, hemolymph MF levels correlated with ovarian development and increased more than 20-fold during 4 days post exposure to adult food (Figure 5). JH-III levels remained relatively low (0.3–2.0 pg/μl hemolymph) throughout the sampling period. JH-IIIB levels remained constant (2.5–3.5 pg/μl hemolymph) for the initial 3 days, but increased to 8.5 pg/μl hemolymph on day 4. MdSGHV infection completely shut down egg development; ovaries from viremic flies remained in the pre-vitellogenesis stage (stage 1). The JH-III and JH-IIIB levels were lower in the viremic than in healthy females throughout the 2–4 d-pi interval. The largest difference in hormone titers was observed in the MF; after 3 d-pi, the hemolymph MF levels in viremic flies were 4- to 10-fold less than those detected in healthy flies.

### Impacts of hormone delivery on viremic female flies

The injection of the PBS-control or virus-injected female flies with either the peanut oil/acetone or the 10% ethanol carriers resulted in ~10 and 30% mortality, respectively, of flies within the 2 d-pi of the carrier (data not shown). Injection of either carrier into PBS-injected females did not impact ovarian/egg development in survivors. Likewise, the survivors of PBS-injected females challenged with JH-III (*n* = 37), MF (*n* = 42), ecdysone (*n* = 35), or the hormone mix had normal ovarian development ranked at stage 4 at 5 d-pi. Hormone challenges with JH-III (*n* = 34), MF (*n* = 41), or ecdysone (*n* = 36) into virus-challenged females did not alter SGH development; at 5 d-pi, all females had SGH symptoms equivalent to virus controls. Furthermore, all surviving females of the hormone + virus treatments displayed ovarian stage 1 development identical to that observed in the virus control females at 5 d-pi.

The impact of ecdysone treatments on modulating the transcription of the two female-specific genes (*Hex2* and *Yo2*) was examined using established qPCR protocols (Lietze et al., 2007). Three d-pi (which equals 3 days on a protein-containing diet), the transcription of hexamerin and yolk protein was reduced 13- and 41-fold, respectively, in protein-fed viremic females when compared with protein-fed healthy females. In sugar-fed healthy females, a 7- and 4-fold reduction of hexamerin and yolk protein transcription was detected when compared with protein-fed healthy females. Injection of ecdysone stimulated both hexamerin and yolk protein transcription in both the sugar-fed and the virus-infected females in an expected, dose-dependent manner (Table 4). Ecdysone amendments induced higher hexamerin transcript levels than the yolk protein transcript levels. When compared with baseline expression levels (1.00) for protein-fed healthy females, the 1 μg ecdysone dose induced a ~10.3- to 9.6-fold upregulation of hexamerin, and a ~1.9- to 1.2-fold upregulation of yolk protein in sugar-fed and virus-infected females, respectively. It should be noted that the ecdysone-induced rescue of these female-specific protein transcripts did not result in egg production in viremic flies.

**Table 4:** **Impacts of ecdysone treatment on modulation of hexamerin-2 (*Hex2*) and yolk-2 (*Yo2*) gene expression in healthy and viremic *M. domestica* females**.

**Infection status**	**Diet**	**Ecdysone dose [μg/μl]**	**Relative expression value**	
			**Hexamerin**	**Yolk protein**
Healthy	Sugar	0	0.15	0.28
Healthy	Sugar	0.1	0.49	0.73
Healthy	Sugar	1.0	10.27	1.87
Healthy	Protein	0	1.00	1.00
Viremic	Protein	0	0.09	0.02
Viremic	Protein	0.1	0.10	0.06
Viremic	Protein	1.0	9.58	1.17

*The experimental flies were subjected to a 3-day sugar or protein diet and challenged with different ecdysone dosages*.

### Impacts of SGHV on AMP levels and on insect-associated microbiota

Both the RNA-Seq data and subsequent RT-qPCR validation assays confirmed that the transcript levels of AMPs were increased by MdSGHV infection; AMPs such as sarcotoxins, defensins, and attacins comprised many of the most highly upregulated genes (Table 2, Figure 3). As expected, insect homogenates prepared from healthy females contained no detectable activity targeting *E. coli* D31 but did contain constitutive lysozyme activity that targeted *M. luteus*. Injections of *E. coli* D31 (positive controls) into females induced antibacterial activity against *E. coli* D31; the relative lysozyme activity was similar to control treatments. Injection of the PBS healthy gland homogenate into female flies induced an immediate anti-D31 activity; however, this antibacterial activity was not sustained and was undetectable by 72 h-pi. Unlike the healthy SG preparations, injection of the MdSGHV SG homogenates induced a sustained antibacterial (D31) activity throughout the 10-day sampling period. Lysozyme activity as measured by the *M. luteus* assay was suppressed initially by the MdSGHV challenge. Insect homogenates prepared from all of the above treatments had no detectable inhibitory activity against either *Serratia marcescens* or *S. cerevisiae* (data not shown). The induction of the innate defense systems by MdSGHV infection and the subsequent increase in the production of antibacterial activity did not appear to impact the cultivable microbiome associated with *M. domestica*. The estimates of CFUs associated with healthy flies (*n* = 32) ranged from 1.5 × 10^5^ to 1.85 × 10^6^ CFUs per fly. The average number of CFUs for both healthy (5.8 ± 4.9 × 10^5^ CFUs per fly) and PBS-injected flies (*n* = 15, 8.0 ± 8.8 × 10^5^ CFUs per fly) was not altered by MdSGHV infection at 72 h-pi. The average number of CFUs from viremic females (*n* = 24) was estimated to be 5.9 ± 8.8 × 10^5^ CFUs per fly. Likewise, the CFU estimates at 96 h-pi were not impacted by virus infection.

## Discussion

### Expression of MdSGHV transcripts

Mapping RNA-Seq reads of the infected libraries onto the MdSGHV genome demonstrated a gradient in the predicted transcription rate of the viral ORFs. Significantly, the RNA-Seq approach proved to be highly sensitive; it detected seven putative ORFs (7, 9, 19, 39, 41, 59, 80, and 106,) undetectable previously by 3′ RACE and qPCR (Salem et al., 2009) and eight ORFs (5, 6, 8, 9, 59, 60, 70, and 106) undetectable in 454 sequencing of a cDNA library from viremic *M. domestica* (unpublished data, Table S7). In general, the transcript frequency of viral ORFs predicted by the RNA-Seq data correlated (*R*^2^ = 0.702) with transcript abundance predicted prior to the 454 reads (Figure S1B). However, predicted transcript frequencies were not associated with known structural features such as ORF position, tandem orientation, 3′-UTR structure, cleavage site, polyadenylation signals, etc., (see Table 1; Salem et al., 2009). However, low-copy ORFs (5, 6, 7, 8, 9, 69, and 60) were clustered in the vicinity of direct-repeat (dr) regions (Figure 1).

Ten of the 15 highly abundant reads detected at 48 h-pi mapped to ORFs encoding for structural peptides (Garcia-Maruniak et al., 2008) (Figure 2; Table S2). These include ORF 96, encoding for the major viral envelope peptide (Boucias et al., 2013a), and ORF 47, a homolog to the occlusion-derived virus envelop protein 66 (ODV-E66), a component of the baculovirus envelope that is important for virus morphogenesis. Notably, *odv-e66* encodes a non-secreted hyaluronate lyase-like enzyme that degrades hyaluronan, a component of the extracellular matrix (Vigdorovich et al., 2007); it is possible that this protein facilitates MdSGHV invasion (i.e., penetration) of host cells. Our annotations using various databases revealed that the MdSGHV086 is a putative viral matrix protein (Table S2). Immuno-cytochemical staining localized the MdSGHV086 protein on the external surface of the nucleocapsids exiting the nuclear pores of infected SG cells (Figure S2). Such viral matrix proteins provide the linkage between the viral envelope and nuclear-core components; they are found in many enveloped viruses (Battisti et al., 2012) and are crucial for viral assembly and budding. The remaining ORFs coding for highly abundant structural ORFs included 40, 71, 85, 23, 28, 97, and 16 (Figure 2); these proteins remained without annotations, except for ORFs 16 and 85, which contained transmembrane domains (Table S2). Taken together, the high number of structural transcripts suggests that at 48 h-pi, viral morphogenesis is well underway, a finding supported by the detection of infectious virus in the salivary secretions of viremic flies at 48 h-pi (Lietze et al., 2009).

The remaining five of the 15 most-abundant proteins are non-structural proteins, of which the most abundant was ORF037, a homolog to a small, nuclear RNA-activating, complex protein involved in TATA box recognition (Table S2). Other non-structural proteins included MdSGHV048, a homolog to ring-infected erythrocyte surface antigen (a virulence factor protein), and MdSGHV082, a site-specific recombinase (RCI)-like protein that possesses a DNA-breaking and -rejoining domain (Alberts, 2003). ORFs 92 and 93 remained without annotations. Other non-structural viral transcripts, which were detected in tissues that did not support viral replication/morphogenesis (Lietze et al., 2011b) and whose relative abundance generally mirrored the RNA-Seq results, included ORFs 1, 10, and 108 (Table S2). MdSGHV010 is homologous to the nudivirus OrNV mitochondrial carrier protein, a protein central to the transport of dATP and dTTP. Mimivirus uses this protein to target the host mitochondria as a source of dNTPs for its replication (Monné et al., 2007). MdSGHV001 is a DNApol-B delta subfamily homolog, which plays a central role in viral genome replication and transcription (Choi, 2012). MdSGHV106 is a PIF-3 homolog, an ODV-specific protein speculated to mediate nucleocapsid translocation along microvilli (Song et al., 2016) and thus facilitate release of nucleocapsids into the cell cytosol to initiate infection. We also quantified expression of ORF MdSGHV108, which encodes a homolog to the baculovirus Ac81-like protein. Ac81 is a late-expressed, non-structural protein of BmNPV, which is thought to interact with the host cellular protein actin A3 (Chen et al., 2007) to facilitate intracellular transport of viral particles and infection.

### Putative functional SGHV proteins

A comprehensive proteogenomic analysis (RNA-Seq and LC-MS/MS) of the two SGHV strains (GpSGHV-Uga and GpSGHV-Eth) infecting the tsetse fly (Abd-Alla et al., 2016) revealed that 60 ORFs encode functional proteins, i.e., the ORFs contained TATA-box/poly(A) signals, had both transcript and peptide mapping, and/or had the G/T/ATAAG late-promoter motifs. Twenty of the MdSGHV proteins had significant homologies to the GpSGHV functional ORFs, 11 of which had RNA-Seq reads of >10,000 (Figure 1; Table S2). These included ORFs 4 (virion protein SGHV082), 13 (nucleocapsid protein SGHV083), 25 (vesicle-associated membrane protein), 29 (PIF-1), 30 (nucleocapsid protein), 33 (cell division protein 48), 55 (casein kinase isoform1-D), 70 (LEF-8), 73 (nucleocapsid protein), 83 (LEF-3), and 84 (glutathione-S-transferase). The remaining nine ORFs had RNA-Seq reads ranging from 4,000 to 10,000. The nine ORFs were: 12 (thymidylate synthase), 36 (MP-NASE), 39 (P74), 74 (LEF-9), 87 (LEF4), 89 (PIF-2), 102 (FAD dependent sulfhydryl oxidase), 107 (ABC transporter), and 108 (Ac81) (Figure 1; Table S2).

### Impacts of MdSGHV infection on host transcriptome

The majority of research conducted on insect DNA virus-host transcriptome interactions has involved the infection studies of susceptible cell lines challenged with high baculovirus titers. In such cases, the virus infection initiates a global shutdown of host transcription (Nobiron et al., 2003; Katsuma et al., 2007). However, studies conducted on host animals do not typically display this event; instead, viral infection displaying tissue-specific tropism occurs in a limited number of host cells. In the case of MdSGHV, viral infection at 48 h-pi downregulated the transcription levels of 1,196 and 2,708 genes at the padj values of 0.01 and 0.2, respectively, out of a total of 17,034 unigenes detected by RNA-Seq. MdSGHV displays a narrow tissue tropism, undergoing detectable levels of morphogenesis in selected gland tissues such as SGs and CA/CC (Lietze et al., 2011b). Prior qPCR analyses demonstrated the presence of viral transcripts and genome copies in samples derived from multiple housefly tissues. Further, electron microscopy revealed the presence of enveloped virus in the cytoplasm of tracheal cells. Here, it should be noted that the tracheal system is integrated into all insect tissues/cells except for the circulating hemocytes. This finding may explain the distribution of MdSGHV genome copies in tissues that do not support viral morphogenesis.

### Regulation of genes related to virus-induced pathologies

To maximize production of viral progeny and to evade/interfere with the host's immune and anti-viral stress responses, DNA viruses dampen the expression of host cell proteins via several strategies (Herbert and Nag, 2016). One strategy is co-transcriptional (in the nucleus) downregulation of the transcription of the host mRNAs such that the host's transcription factors and RNA polymerase complex components are availed for viral replication. In this strategy, viruses may encode proteases (to degrade) or other proteins (to inhibit) transcription factors of the host insect. From our study, the housefly transcription initiation factor II D (TFIID) was among the downregulated proteins (log2FC = −6.0) (Table S3). Interestingly, MdSGHV037, which had the most RNA reads (See Figures 1, 2), encodes a homolog to the small nuclear RNA activating protein 190 (SNAP190), a TATA-box binding protein that may preferentially lead to transcription of viral genes. Probably this protein out-competes the downregulated housefly TFIID such that MdSGHV genes are preferentially transcribed by the host machinery.

Viruses also maximize their replication post-transcriptionally (in the nucleus or cytoplasm) via decapping or degradation of host mRNAs (Narayanan and Makino, 2013) and via interfering with mRNA splicing and nuclear export. The virally encoded decapping enzymes are usually expressed during early and late stages of viral infection to target host mRNAs such that host translation machinery is available almost exclusively for translation of viral RNAs (Liu et al., 2015). However, none of the MdSGHV genes had any significant homologies to known viral decapping enzymes. Alternatively, to promote replication, dsDNA viruses encode their own capping enzymes to protect their mRNAs (Decroly et al., 2012). The highly transcribed MdSGHV087 is a LEF-4-like protein (Figures 1, 2; Table S2), which is putatively functional in GpSGHV (Abd-Alla et al., 2016); LEF-4 is an mRNA-capping protein essential for baculovirus replication (Jin et al., 1998).

Nuclear replicating DNA viruses must deliver/export their genomes into and out of the host cell nucleus, a process orchestrated via the tightly regulated traffic through the nuclear pore complex (NPC) (Fay and Panté, 2015). Depending on the virus, the nuclear transmission of nucleocapsids involves specific transport mechanisms to import/export the viral genome (and associated proteins) through the NPC. To allow traffic of the viral nucleoprotein complexes across the NPC, one would expect degradation of nuclear pore proteins and removal of the nuclear basket that controls active NPC functions. Some viruses induce activation of nuclear factor NF-κ-B, which in turn results in auto-activation of cellular caspases that promote viral nucleoprotein complex export (Mühlbauer et al., 2015). Prime caspase substrates include nuclear pore complex protein-153 (Nup153) and lamins (Fischer et al., 2003), two important NPC nuclear basket components and key players in nuclear import/export control. This potentially accounts for the upregulation of caspases-3/-8, lamins, and NF-κ-B p110 subunits, as well as the downregulation of Nup153 in MdSGHV-infected samples (see Table S4).

To limit viral replication and protect the host's genome, infected cells express components of DNA damage response (DDR) pathways, which are responsible for detection and repair of DNA lesions; DDR ultimately induces apoptosis (Weitzman et al., 2010). This possibly partially explains the modulation of proteins of the phosphatidylinositol signaling system in the MdSGHV samples (see Tables S5, S6). Some viruses respond by synthesizing anti-apoptotic proteins that allow persistent viral infections. In addition to conferring selective advantages to the virus, viral interference with apoptosis plays essential roles in cellular transformations such that the cells not only survive but also simultaneously grow and efficiently produce the progeny virus (Thomson, 2001). It is therefore noteworthy that MdSGHV078 encodes (moderately; Figure 2) IAP, a potent and specific inhibitor of caspase-3 capable of reversing mitochondrial membrane permeabilization (MMP) (Crook et al., 1993); MMP is a key event in the induction of apoptosis.

Upon activation, DDR-associated kinases phosphorylate multiple substrates, including histones and chromatin-remodeling complexes (Van Attikum and Gasser, 2009). Ultimately, the DDR signaling results in cell-cycle arrest that allows either DNA repair or apoptosis. The virus-induced blockade of apoptosis, coupled with enhancement of virus replication and cellular transformations, undoubtedly not only promotes host genomic instability but also contributes to viral pathogenesis. DNA viruses are able to disable some antiviral facets of the DDR (Weitzman et al., 2010), including repression of histone/chromatin modifications, to render the host cells more favorable to viral DNA replication. This probably explains the downregulation of at least six components of the forkhead box O (FoxO) signaling pathway in the MdSGHV-infected samples, including the serine/threonine-protein kinase ATM (a DDR) (See Table S5). It should be noted that since FoxO is primarily responsible for the maintenance of cellular metabolic stress (Eijkelenboom and Burgering, 2013), FoxO may engage apoptosis if the virus-induced cellular damages become excessive. FoxO is also implicated in epigenetic regulation of gene expression (Calnan and Brunet, 2008).

Under the stress of virus infections, cells must maintain normal homeostasis; otherwise, they die. The main homeostatic adaptive cellular responses are hypertrophy (enlarged cells incapable of dividing), hyperplasia (enlarged cells capable of replication), atrophy (shrinkage) and metaplasia (reversible replacement of an adult cell type with another). To accommodate the additional functional demands for replication of viral progenies, the MdSGHV induces both nuclear and cellular hypertrophy, resulting in a non-lytic increase in individual cell sizes (without cell number increase), and ultimately, enlarges infected glands. Alternatively, the GpSGHV, which causes limited cellular hypertrophy, induces cellular hyperplasia of the SG cells (Kariithi et al., 2013).

### MdSGHV-induced female sterility

The major outcome of MdSGHV infection is female sterility, i.e., inhibition of ovarian development in young flies and subsequent gonadotropic cycles in older females (Lietze et al., 2008). RT-qPCR and present RNA-Seq data clearly showed that virus infection blocks production of the female-specific yolk proteins (YPs) and female hexamerin, which serve as the nutritional basis for egg development (Moreira et al., 2009; Siegenthaler et al., 2010). The YPs synthesized in the fat body are released into the hemolymph and sequestered by the developing basal oocytes for subsequent yolk formation (Dong et al., 2009). In the RNA-Seq experiments, the virus-induced suppression of these female-specific gene transcript levels was not as pronounced as had been found in an earlier experiment, when at 3 days post-infection, viremic flies contained 94-fold and 1,596-fold fewer hexamerin and yolk protein transcripts, respectively, than did control flies (Lietze et al., 2008). A possible explanation may be due to sampling, because hexamerin and YP production fluctuate during the gonadotropic cycle, or to variance between the RT-qPCR and RNA-Seq analyses.

In dipterans, oogenesis is regulated by a hormonal regime (sesquiterpenoids and steroids) that is initiated when emerging flies ingest a protein meal whereby a pulse of JH makes the fat body competent for YP synthesis (Agui et al., 1985). The JH synthesis, involving 13 discrete enzymatic steps (Noriega, 2014) takes place in the CA. Our results gave evidence that the housefly CA/CC supports limited MdSGHV morphogenesis (see Figure S3). In houseflies, JH positively regulates YP levels; allatectomy abolishes YP production (Adams et al., 1985). Our RNA-Seq data sets did not provide clear evidence of blockage of the JH pathway; rather, several of the JH enzymes were variously but insignificantly modulated in the MdSGHV-infected flies. However, chemical analysis of hemolymph revealed that sequesterpenoid levels in viremic females were lower than those detected in healthy flies. Whether or not these differences have a physiological impact on modulating vitellogenesis is unknown. However, amending virus-infected females with exogenous hormone treatments (including JH-III, MF) did not result in normal oogenesis, suggesting that the observed reproductive block was not due solely to a lack of sesquiterpenoids.

An alternative target may be downstream of the JH biosynthetic pathway. The JH-resistant *Methoprene-tolerant* (*Met*) gene detected in the red flour beetle *T. castaneum* (Konopova and Jindra, 2007) is believed to play a critical role in insect morphogenesis. It is thought that JH is an activating ligand for Met (Jindra et al., 2013). Met requires its interacting partner Taiman (Tai) for proper functioning. In mosquitoes, JH acts via Met/Tai to regulate expression dynamics of thousands of genes during female reproduction (Zou et al., 2013). Interestingly, both a homolog to the *Drosophila Met* gene was downregulated (log2FC = −1.9) and several *Drosophila* Tai homologs were moderately downregulated (log2FC = −1.7 and −0.9) in MdSGHV-infected flies.

It should be emphasized that in *M. domestica*, the main controlling agent of YP synthesis is 20-E pulse, which is produced by the ovarian follicular epithelium (Adams et al., 1985). Cytochrome P450 (CYP450) enzymes are involved in the ecdysteroid metabolic pathways (Iga and Kataoka, 2012) and are transcriptionally regulated to support the high biosynthetic activities occurring during ecdysteroid-mediated pulses that trigger molting (Rewitz et al., 2006). In the current study, several components of the CYP450 transcripts were variously modulated in the MdSGHV-infected samples. These included CYP304a1, 307a1, 313a4, 18a1, 28d1, CYP12a2-like, 9f2, 6d1-like, 4e2-like, 6a14, and 6a21. The levels of these gene transcripts are responsible for regulating the ecdysone titer. The downregulated CYP307a1 (Spook), responsible for converting 22, 2-dideoxyecdysone to 2-deoxyecdysone, is a stage-specific regulator for ecdysteroid synthesis in insects (Gilbert, 2004; Ono et al., 2006), whereas others, such as CYP18a1, are implicated in the inactivation of 20-E. Hormone treatment of viremic females with exogenous ecdysone did upregulate the transcription of female-specific genes (Yp, hexamerin) but did not result in normal egg development.

### MdSGHV-induced female behavior changes

In addition to shutting down oogenesis, MdSGHV infection also causes perturbation in housefly mating behavior, specifically the refusal of infected females to mate with either healthy or viremic male flies. In healthy virgin flies, mating behavior is a fixed pattern, initiated by males who mount and stroke/caress the female, stimulating her to extend her ovipositor into the male genital opening, resulting in a copulation event lasting for about 60 min (Murvosh et al., 1964). Infected females, however, respond to male courtship but refuse to extend their ovipositor, thus terminating the mating sequence. From our RNA-Seq data, homologs to the *Drosophila* sex lethal (Sx1), fruitless (Fru), dissatisfied (Dsf), transformer-2 (Tra-2), and doublesex (Dsx), were detected, but only Tra-2 (Log2FC = −1.00) had a padj value of <0.01. However, the conclusions that can be drawn from these data are limited; transcripts of these genes could be better resolved by analyses of tissue-specific RNA-Seq libraries. This qualification notwithstanding, the insect's mating behaviors are also controlled by other hormones, including sesquiterpenoids. In fact, it has long been established that receptivity of virgin males for mating is under the control of JH; Loher and Huber (1966) reported that removal of corpora allata from newly-eclosed grasshopper females resulted in sustained active rejection of courting males. On the other hand, by injecting JH, the authors were able to induce the allatectomized females to accept courting males. It also has been demonstrated that rising hemolymph JH titers stimulate other behavioral traits, including host-seeking in newly-eclosed mosquitoes. For instance, it has been reported that JH activates Met protein in the formation of a transcription repressor complex with proteins Hairy and Groucho; the interactions between these proteins is essential for female maturation (Jindra, 2016), and consequently influences the mating behavior. In our RNA-Seq data, we detected a *Drosophila* homolog to protein Groucho, which was downregulated, albeit at low level (log2FC = −0.5). On the other hand, protein Hairy, which is downstream protein Met, was slightly upregulated in our study (log2FC = 0.2).

### Impact of MdSGHV infections on housefly innate defense systems

Insects have three main antiviral defense mechanisms, one of which is the first antiviral defense line mediated by the siRNA arm of the RNAi pathways (Ding, 2010). Insects encode dicer-1 (Dcr-1) and Dcr-2 to generate and activate siRNAs, respectively; Dcr-2 directly links the siRNA pathway to the Jak/STAT pathway (via the siRNAs) (Paradkar et al., 2012). Argonaute-2 (Ago-2) executes siRNA-mediated gene silencing (Okamura et al., 2004). Interestingly, Ago1 was downregulated by MdSGHV infection; Ago-1 is important in production of miRNAs that are crucial to oogenesis (Nakahara et al., 2005; Azzam et al., 2012). Disruption of *LmAgo-1* by RNAi dramatically decreased vitellogenin transcription and severely impaired follicular epithelium development, terminal oocyte maturation, and ovarian growth, supporting the notion that Ago1-dependent miRNAs are required for JH-mediated vitellogenesis and egg production in locusts (Song et al., 2013). From our RNA-Seq data, a homolog to the *Drosophila* Dcr-2 was upregulated (log2FC = 2.9), as well as two Ago-2 homologs (log2FC = 2.1 and 3.9) in infected flies, implying that siRNA pathway is an important antiviral immune response in houseflies. However, the RNA-Seq did not provide any evidence that MdSGHV encodes viral suppressors of siRNA. Potentially, MdSGHV could have evolved other RNAi counter-defense mechanisms, such as sequestration in cytoplasmic compartments, which may be inaccessible to the host's RNAi machinery.

The siRNA antiviral response does not necessarily clear viral infections, which brings into play the innate antimicrobial (Jak/STAT) and NF-κ-B innate immune pathways (Imd and Toll) (Kingsolver et al., 2013). One of the Jak/STAT pathway components, which was moderately downregulated (log2FC = −1.2) in MdSGHV-infected flies, is tyrosine-protein kinase hopscotch. Hopscotch transphosphorylates STATs, which are subsequently transported to the nucleus to regulate expression of downstream effectors of the pathway. Perhaps downregulation of hopscotch could be a strategy of MdSGHV to disrupt the phosphorylation and/or nuclear export of STATs. The Jak/STAT pathway ligand, Vago protein (Paradkar et al., 2012), whose expression is Dcr-2-dependent, was moderately upregulated (log2FC = 1.5) in the infected flies. Moreover, the virus-induced stress in insects results in upregulation of other Jak/STAT downstream effectors such as the thioester-containing proteins (Teps), which mediate phagocytotic antiviral responses (Levashina et al., 2001). Notably, our RNA-Seq revealed various levels of upregulation (log2FC of between 1.1 and 3.4) of *Drosophila* and *Glossina* Tep-1, 2 and −4 homologs. In terms of the NF-κ-B, the Imd and Toll activate cellular transcription mediated by Relish and NF-κ-B orthologs (the terminal transcription factors for Imd) and Dorsal/Dorsal-related proteins (Dif; for the Toll pathway). The two isoforms of the housefly NF-kappa-B p110, which were upregulated in MdSGHV-infected flies (log2FC = 1.6), are *Drosophila* Relish homologs. The two Toll transcription factors also are centrally involved in early embryogenesis, which possibly explains the moderate downregulation of Dorsal homologs in the infected flies, including two isoforms of embryonic polarity protein dorsal-like protein (log2FC = −2.4 and −0.9) and dorsal-interacting protein 3 (Dip3; log2FC = −2.1). We also noted upregulation of various components of Toll pathway signaling in the infected flies, including PGRP-SD/SA, GNBP-3, and Cactus.

An alternative antiviral defense mechanism is the autophagy pathway, a highly conserved cellular degradative pathway, which is induced by among others ER stress, ROS and mitochondrial damage triggered by pathogen infection (Chiramel et al., 2013). Autophagy is independent of the Jak/STAT, Imd and Toll pathways, and is implicated in the elimination of intracellular pathogens (Yano et al., 2008; Nakamoto et al., 2012). As an antiviral machinery, the autophagy system, which is mediated via autophagy-related genes (Atgs), not only directly degrades viral particles (autophagosomal degradation), but also delivers viral components to Toll receptors on endosomes (lysosomal degradation), and transports host's antiviral proteins to virus replication sites (to interfere with viral replication) (Yordy et al., 2013). Some of the key proteins involved in autophagy include microtubule-associated proteins (MAPs), γ-aminobutyric acid receptor-associated proteins (GABARAP). From out RNA-Seq data, *Map-2*-like and *Gabarap*-like genes were moderately upregulated (log2FC = 2.2 and 3.8, respectively) in viremic compared to healthy flies. In *Drosophila*, the autophagy pathway is mediated via the phosphotidylinositide-3-kinase (PI3)/Atk/mTOR pathway; knock-down of autophagy-related genes increased virus production in both *Drosophila* S2 cells and in the flies (Shelly et al., 2009). From our RNA-Seq data, *Drosophila* PI3K/mTOR homologs were slightly downregulated in the MdSGHV-infected flies, including PI3K59F (log2FC = −0.2), Pi3K68D (log2FC = −0.3), (log2FC = −0.4), (log2FC = −0.6) and an mTOR-like protein (log2FC = 0.1). Further, it has been shown that some dsDNA viruses have over evolutionary time developed tactics not only to evade, but also to exploit the autophagy system to the advantage of viral replication (See reviewed in Chiramel et al., 2013). Various pathways such as the above-mentioned Akt/mTOR signaling pathways are involved (Shoji-Kawata and Levine, 2009).

A major outcome of MdSGHV infection is the higher transcription level of a cocktail of AMP genes, resulting in sustained increase in antibacterial cationic peptide activities. Complementing the cationic peptides is the increased transcription of various PGRPs and lectins, molecules that serve as sentinels in the non-self recognition of invasive bacteria and regulators of commensal gut bacteria (Royet et al., 2011). However, the increase in AMP and PGRP activities did not impact the cultivable microbiome associated with the host; total CFUs in viremic flies were comparable to those estimated for healthy flies. Here, it should be noted that the housefly microbiome is localized on the cuticle and in the lumen of the digestive tract; hence, the microbiome may not be exposed to AMPs affiliated with internal tissues. Furthermore, the ability of the microbiome to coexist with the house flies lab colony may be due intrinsic resistance to AMPs. Moreover, MdSGHV is non-lytic, replicates in limited tissues (SG, CA), and causes a chronic nonlethal disease. The induction of innate defenses by infection may underlie the tissue tropism of this virus and/or may serve to suppress secondary infections, thereby assuring the continued production and release of virus from viremic flies.

## Conclusions and future perspectives

The RNA-Seq analysis demonstrated that the MdSGHV infection caused a massive alteration in host gene transcription. Active MdSGHV replication occurred at 48 h-pi as evidenced by the abundant levels of MdSGHV transcripts. Annotation of viral genes was associated with various facets of viral morphogenesis, including host cell invasion/penetration, nucleocapsid cytoplasmic traffic, genome replication, assembly, SGH-induction (virulence factors), and egress (budding). The multiple fronts employed by MdSGHV, including downregulation of host transcription factors, epigenetic gene expression regulation, production of virally encoded capping enzymes and blockade of apoptosis, significantly contributes to SGH development in houseflies. It would be interesting to make a comparative analysis of *Glossina*-GpSGHV and *Musca*-MdSGHV models, especially to identify host factors involved in the development (or lack thereof) of diagnostic SGH symptoms.

MdSGHV transcription/infection caused a massive reduction of transcript levels of genes associated with female reproduction/egg development; these findings confirmed prior observations on MdSGHV-induced sterility. The gene product(s)/pathway(s) that is responsible for oogenesis shutdown is unclear. Our speculation was that limited infection of CA/CC-complex may alter hormonal titers required to upregulate vitellogenesis/egg production. Our hormone therapy attempts, although rescuing the transcription of female-specific proteins, did result in egg production. It is possible some of the “down regulated” genes such as the *Ago-1* or *Spook* homologs may prevent the upregulation of the multiple genes/pathways involved in ovary/egg maturation. Potentially, the MdSGHV-mediated modulation of hormones may explain the refusal of viremic females to mate; the CA/CC-produced hormones mediate not only insect morphogenesis, ovary maturation, and general physiology but also modulate mating behavior. A key to understanding the observed sterility is identifying the viral gene product(s) that triggers this event. However, it is not easy to unravel the roles of specific, virally encoded factors (proteins) at the molecular level; most MdSGHV proteins have no identifiable homologs. Potentially, testing recombinant viral gene products in female houseflies or expressing gene constructs in heterologous hosts such as *Drosophila* may elucidate function, setting the stage for the discovery of a novel viral-gene-based insect birth control. Additional studies should address the full repertoire of players downstream in the JH pathway.

MdSGHV infection resulted in the increased transcript levels of genes associated with innate defense pathways. One may speculate that the observed increase is induced by virus infection/replication. Limited bioassays have demonstrated that the translation of these genes results in increased levels of AMPs. The induction of these pathways may potentially provide these viremic insects protection against opportunistic pathogens or serve to restrict the infection to select glandular tissues. It is important, however, to note that the interpretations of the innate defense responses were complicated by the fact that the experiments were conducted on the whole animal and not a cell culture. The physiological status of the healthy female fly is dynamic; for instance, access to a protein meal is known to trigger a massive physiological switch that is directed at egg production (Attardo et al., 2005). It has been proposed that in insects, there are reproduction-immunity trade-offs in resource allocations (Schwenke et al., 2016); these trade-offs are mediated by the endocrine and metabolic signaling, which heavily rely on the fat body. Critical of these resources include yolk proteins, vitellogenins, lipid, RNAs and ribosomes, which are allocated intricately for egg production reduces immune response and alternatively the induction of the innate defense system decrease reproductive fitness. Therefore, many of the pathways that were apparently upregulated could be the result of MdSGHV infection to block reproduction, and thus, at 48 h-pi, the viremic females are reminiscent of individuals that are more comparable to newly eclosed females.

## Author contributions

DB conceived the study design, conducted the infection assays, RNA extractions, diagnosis, data compilation, and wrote various sections of the manuscript. HK performed gene annotation, pathway analysis, data compilation, integration of data sets, and wrote the major sections of the manuscript. XY conducted and analyzed the RT-qPCR reactions. FY conducted the compilation and initial annotation the RNA Seq data set. PT conducted sequesterpenoid HPLC analysis. CV conducted fly injections, RNA extractions, PCR reactions, antimicrobial bioassays and bacterial CFU determination. All the authors read and approved the final version of the manuscript.

## Funding

This work was financially supported by a University of Florida Multistate Minigrant, and by the Joint FAO/IAEA (CRP #D42015). Division of Nuclear Techniques in Food and Agriculture, Vienna, Austria.

### Conflict of interest statement

The authors declare that the research was conducted in the absence of any commercial or financial relationships that could be construed as a potential conflict of interest.

## Supplementary material

The data discussed in this paper have been deposited in the NCBI's Gene Expression Omnibus (GEO) (Edgar et al., 2002), and are accessible through GEO Series accession number GSE88939 (https://www.ncbi.nlm.nih.gov/geo/query/acc.cgi?acc=GSE88939).

Figure S1 **Mapping of RNA-Seq reads onto the MdSGHV genome**. The figure shows a regression analysis of the size and RNA-Seq read frequency of all the 108 ORFs in the MdSGHV genome **(A)** and a comparison between the frequency of the RNA-Seq reads and prior 454 data set **(B)** (see Table S7).

Figure S2 **Immuno-localization of MdSGHV086 using mono-specific anti-MdSGHV086 IgG**. The antibody was generated in rabbits using an antigen-derived recombinant MdSGHV086. **(A)** Western blot probed with anti-MdSGHV086 antibody: lane 1 pre-stained MW standards, lane 2 healthy gland homogenate, lane 3 infected gland displaying SGH, and lane 4 gradient purified MdSGHV. **(B)** TEM micrographs of thin section of hypertrophied SGs probed with anti-MdSGHV086 antibody and a commercial anti-rabbit colloidal gold secondary probe. The MdSGHV086 was localized on the surface nucleocapsid being synthesized in the virogenic stroma of the infected nucleus. **(C)** Immuno-staining of nucleocapsids exiting through the nuclear pores of infected SG cells. **(D)** Enveloped virus in the gland lumen, note the antibody probe appeared to localize between the capsid and outer envelope region.

Figure S3 **TEM micrographs of the CA/CC complex dissected from infected females at 48h-pi. (A)** Depicts a cell containing a hypertrophied nucleus with condensed chromatin reminiscent of that observed in infected SG cells. **(B)** Is a higher magnification of this region showing the presence of numerous elongate MdSGHV nucleocapsids providing evidence for limited viral morphogenesis in the CA/CC complex.

Table S1 **Description of host genes and PCR primer pairs**. Primer sets designed from ORFs from *Musca domestica* genes that displayed varying degrees of regulation of MdSGHV infection.

Table S2 **Annotations of 108 proteins in the MdSGHV genome**. Annotations of 108 proteins in the MdSGHV genome: Descriptions of the best BLASTp homologies to the known viral and/or cellular genes are indicated (Bit Score ≥ 40; Expect value of E-6). The signature domains in the protein sequences are indicated in the last column. Amino acid coordinates of the N-terminal signal peptide (SP) sequence and transmembrane domains (TM) are indicated in the last column. Of the 108 MdSGHV proteins, 31 proteins are putative transmembrane proteins as they contained at least one TM. ORFs marked with asterisks (^*^) denote 10 of the most abundant MdSGHV structural proteins based on the RNA-Seq reads at 48 h-pi (Compare with Figure 2). The 20 MdSGHV ORFs in bold encode proteins that ate homologous to proteins in the two SGHVs infecting the tsetse fly (GpSGHV-Uga and GpSGHV-Eth; Abd-Alla et al., 2016), which have been reported to be functional proteins [i.e., contain TATA-box, poly(A) signal sequences and have been confirmed by RNA-Seq and LC-MS/MS].

Table S3 **Gene Ontologies (GO) of downregulated host proteins in viremic houseflies**. Annotations and description of the GO terms associated with the top 50 genes (padj ≤ 0.01) in the housefly that were negatively regulated by MdSGHV infection at 48pi. For the annotations, the translated protein sequences were initially blasted using BLASTp Blast2GO (Conesa et al., 2005). Further annotations were performed at the nr-NCBI database (Bitscore ≥ 80; *E*-value ≤ 1.0E-6) on *Drosophilidae* database. A total of 14 uncharacterized housefly sequences (marked with asterisks) yielding uncharacterized *Drosophila* homologs were assigned the closest characterized dipteran homologs available at the nr-NCBI database.

Table S4 **Gene Ontologies (GO) of upregulated host proteins in viremic houseflies**. Top fifty genes having a padj value of ≥0.01 positively regulated by MdSGHV infection at 48pi. Gene information derived from annotation to *M. domestica or Drosophila* databases. For the annotations, the translated protein sequences were initially blasted using BLASTp Blast2GO (Conesa et al., 2005). Further annotations were performed at the nr-NCBI database (Bitscore ≥ 80; *E*-value ≤ 1.0E-6) on *Drosophilidae* database. The seven uncharacterized housefly sequences (marked with asterisks) yielding uncharacterized *Drosophila* homologs were assigned the closest characterized dipteran homologs available at the nr-NCBI database. Five sequences remained without any significant hits to characterized proteins in the databases.

Table S5 **Pathways associated with upregulated proteins**. KEGG pathway mapping of *Musca domestica* genes whose transcription was upregulated in the MdSGHV-infected flies compared to the PBS-injected controls. The numbers of the host genes that were upregulated are shown in reference to the total number of housefly genes that have been associated with each pathway. The KEGG pathways are hyperlinked.

Table S6 **Pathways associated with downregulated proteins**. KEGG pathway mapping of *Musca domestica* genes whose transcription was downregulated in the MdSGHV-infected flies compared to the PBS-injected controls. The numbers of the host genes that were downregulated are shown in reference to the total number of housefly genes that have been associated with each pathway. The KEGG pathways are hyperlinked.

Table S7 **Comparative transcriptome analysis**. Comparison of the frequency of RNA-Seq reads of the MdSGHV infected libraries (V1, V2, and V3) mapped to all the 108 ORFs in the viral genome to the previously 454 reads generated from cDNA generated from a total RNA extracted from a pool of flies sampled at 2, 4, and 6 d-pi.
