# Supplementary figures and images for: Responses of the Housefly, Musca domestica, to the Hytrosavirus Replication: Impacts on Host's Vitellogenesis and Immunity
Source: Front Microbiol. 2017 Apr 5;8:583. doi: 10.3389/fmicb.2017.00583 (PMC5380684; doi:10.3389/fmicb.2017.00583)

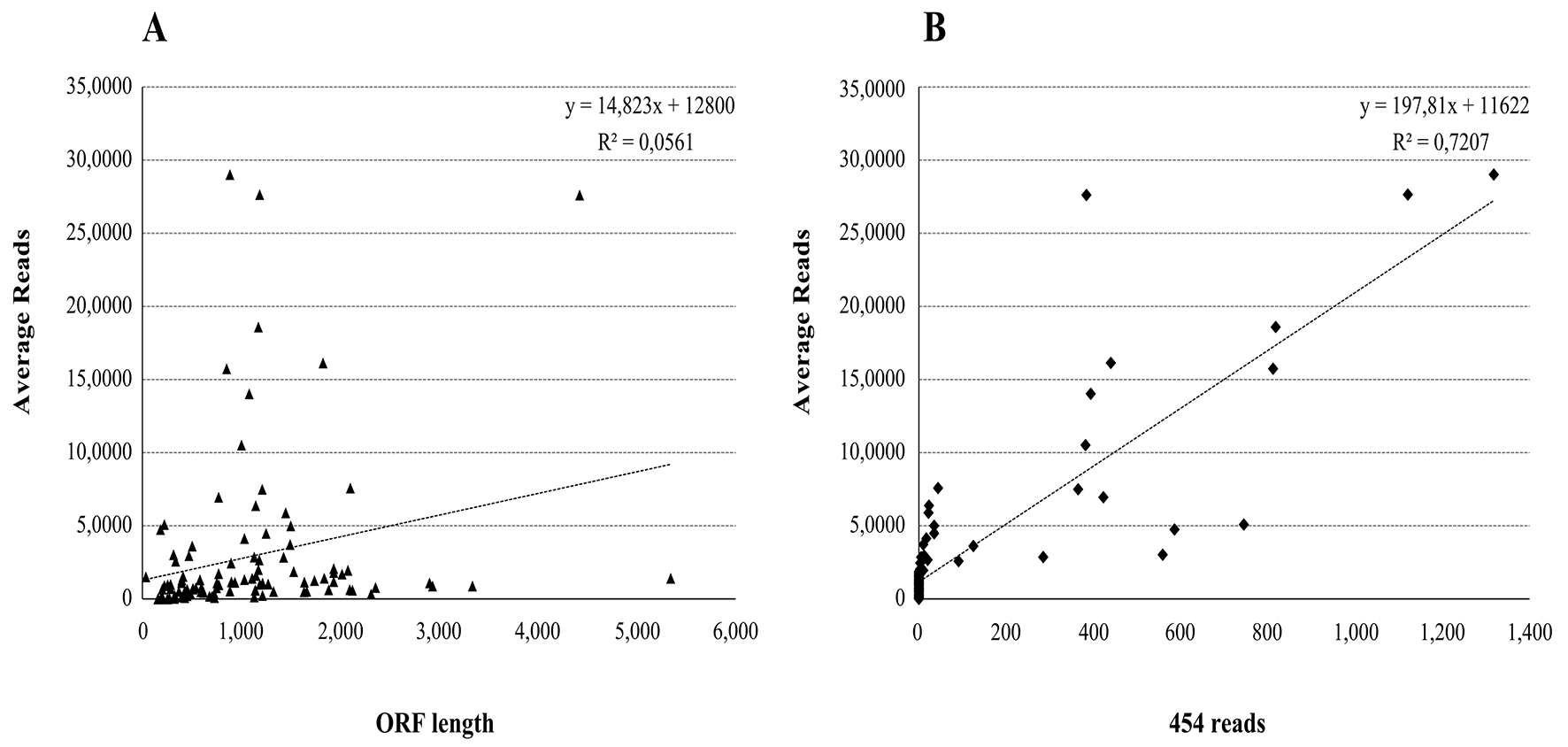

Supplement: Figure S1 — Mapping of RNA-Seq reads onto the MdSGHV genome. The figure shows a regression analysis of the size and RNA-Seq read frequency of all the 108 ORFs in the MdSGHV genome (A) and a comparison between the frequency of the RNA-Seq reads and prior 454 data set (B) (see Table S7). [file Image1.TIF]

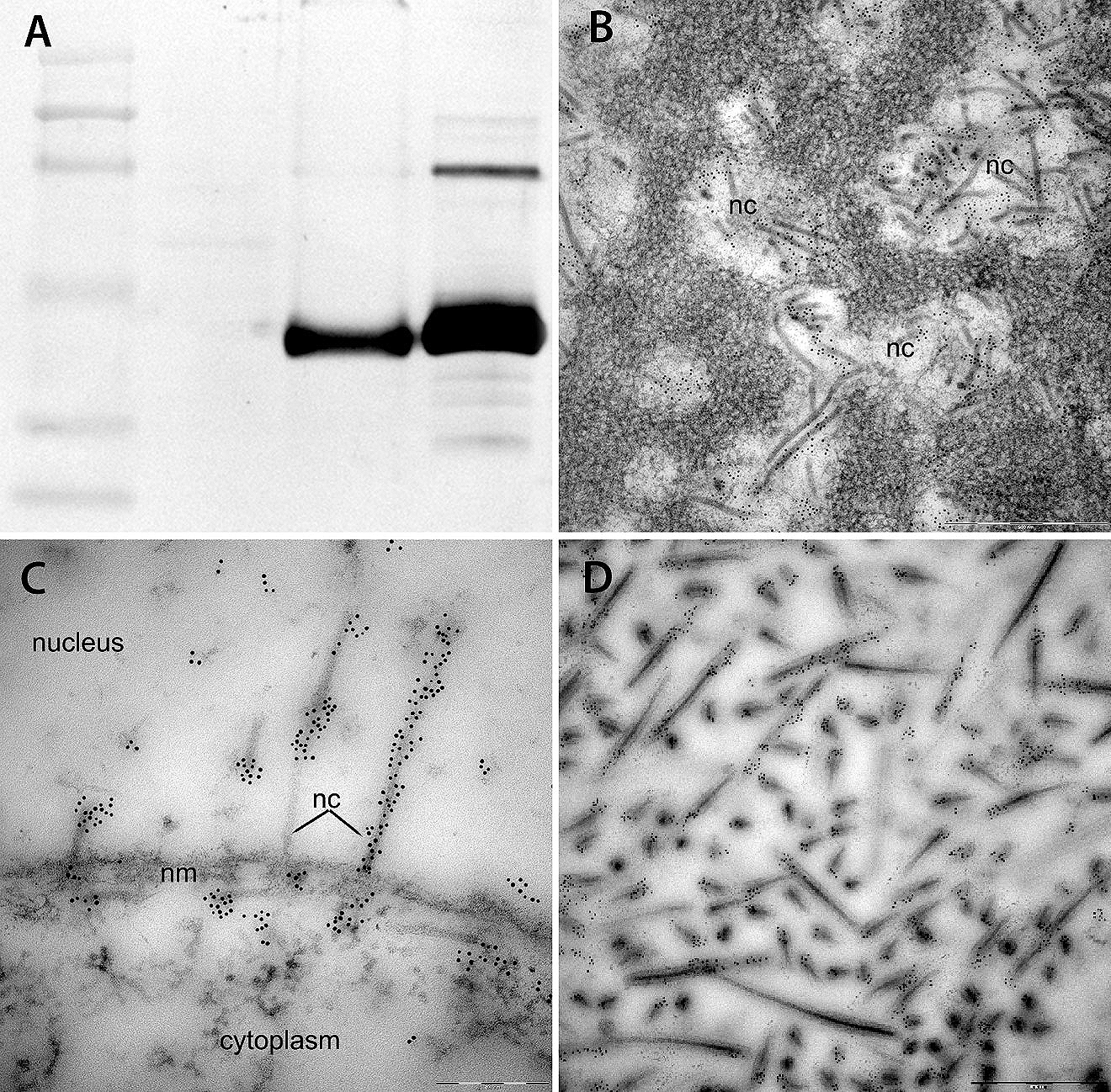

Supplement: Figure S2 — Immuno-localization of MdSGHV086 using mono-specific anti-MdSGHV086 IgG. The antibody was generated in rabbits using an antigen-derived recombinant MdSGHV086. (A) Western blot probed with anti-MdSGHV086 antibody: lane 1 pre-stained MW standards, lane 2 healthy gland homogenate, lane 3 infected gland displaying SGH, and lane 4 gradient purified MdSGHV. (B) TEM micrographs of thin section of hypertrophied SGs probed with anti-MdSGHV086 antibody and a commercial anti-rabbit colloidal gold secondary probe. The MdSGHV086 was localized on the surface nucleocapsid being synthesized in the virogenic stroma of the infected nucleus. (C) Immuno-staining of nucleocapsids exiting through the nuclear pores of infected SG cells. (D) Enveloped virus in the gland lumen, note the antibody probe appeared to localize between the capsid and outer envelope region. [file Image2.TIF]

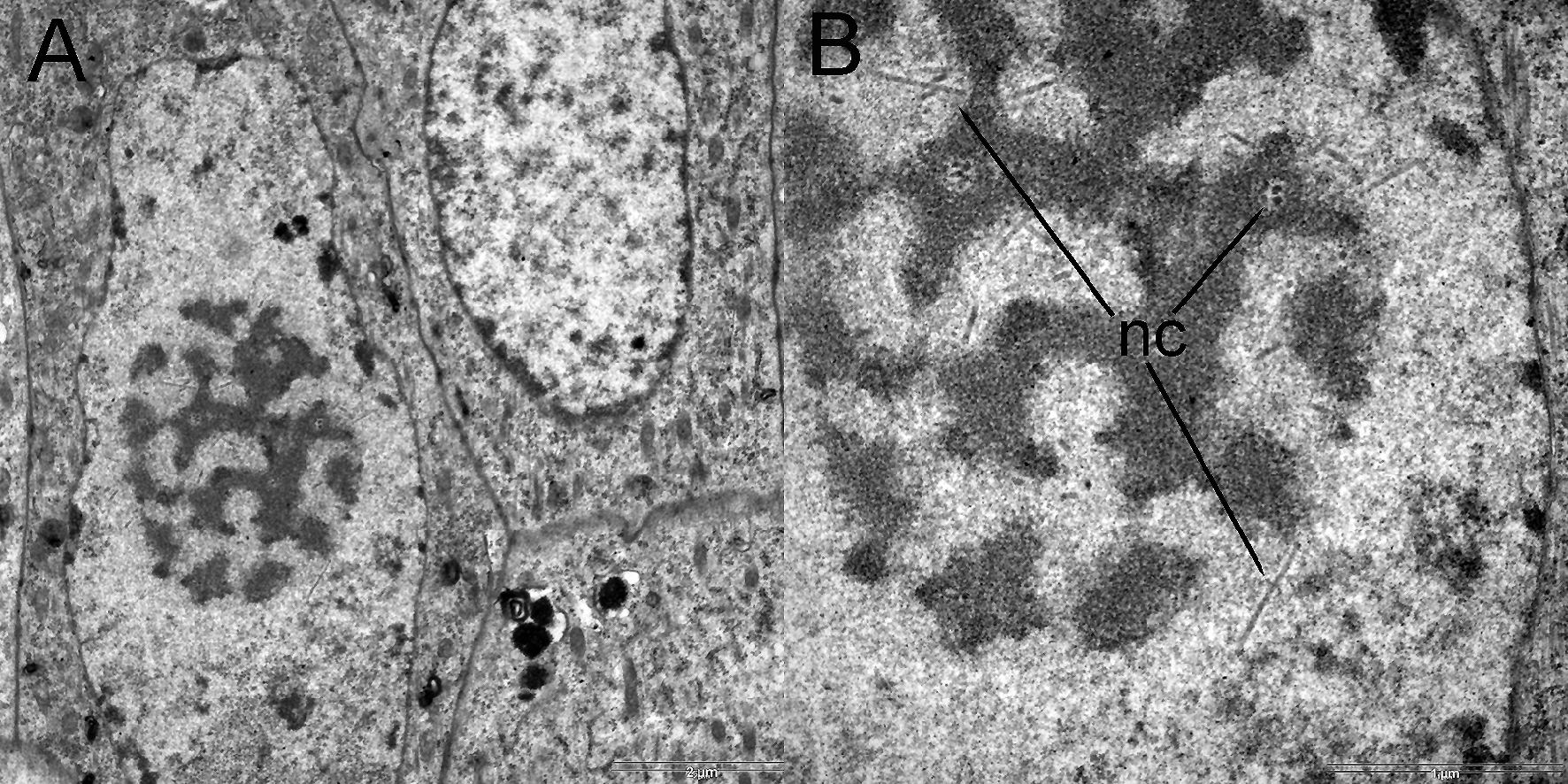

Supplement: Figure S3 — TEM micrographs of the CA/CC complex dissected from infected females at 48h-pi. (A) Depicts a cell containing a hypertrophied nucleus with condensed chromatin reminiscent of that observed in infected SG cells. (B) Is a higher magnification of this region showing the presence of numerous elongate MdSGHV nucleocapsids providing evidence for limited viral morphogenesis in the CA/CC complex. [file Image3.TIF]
