# Supplementary material for: Responses of the Housefly, Musca domestica, to the Hytrosavirus Replication: Impacts on Host's Vitellogenesis and Immunity
Source: Front Microbiol. 2017 Apr 5;8:583. doi: 10.3389/fmicb.2017.00583 (PMC5380684; doi:10.3389/fmicb.2017.00583)
Supplement: Table S1 — Description of host genes and PCR primer pairs. Primer sets designed from ORFs from Musca domestica genes that displayed varying degrees of regulation of MdSGHV infection. [file DataSheet1.DOCX]

Supplementary Material

Responses of the Housefly, *Musca domestica*, to the Hytrosavirus Replication: – Impacts on Host’s Vitellogenesis and Immunity

Henry M. Kariithi, Yao Xu, Fahong Yu, Peter E. Teal^[[1]](#footnote-1)^†, Chelsea P. Verhoeven, Drion G. Boucias

**Correspondence:** D. G. Boucias, Entomology and Nematology Department, University of Florida, 970 Steinmetz Hall, Gainesville, Florida 32611, USA. E-mail: [pathos@ufl.edu](mailto:pathos@ufl.edu)

# Supplementary Tables and Figures

## Supplementary Tables

**Table S1. Description of host genes and PCR primer pairs.** The primer sets were designed from ORFs from *Musca domestica* genes that displayed varying degrees of regulation of MdSGHV infection.

| **Identity** | **Description of the gene** | **Forward primer (5'- to -3'** | **Reverse primer (5'- to -3'** |
| --- | --- | --- | --- |
| LOC101892105 | Argonaute-2 | ACAAACCGATGAGAGCCTTG | CACGTGGAAATGACTTGTGG |
| LOC101890924 | Argonaute-2a | ATTCAAAGCACCGCCAAACC | AACAGATCGGTTGCAACGTG |
| LOC101893656 | Dicer-1 | AGCAGCAGGTGCGAATCTAT | GCATAAGTGGTGGGCTGTCT |
| LOC101897503 | CD63 antigen | ATTCCTGTTGCGGTACCTTG | GTTCTACAAGCGCAACACCA |
| LOC101889135 | Peritrophin-1 | AAACTGATACGCCCGACAAG | GGCCACAATGGTACACACAA |
| LOC101900447 | Mucin-19 | ATGGCGAAGAGATGATGACC | GAACAATGGTGCTGGTGTTG |
| LOC101891911 | Multidrug resistance-associated protein 4 | TGGCCAAGAAGAGGATATGG | CACCTTCTCCAACCAGGGTA |
| LOC101890610 | Proline-rich protein 4 transcript variant X1 | GTCTTGCCACATTGTTGGTG | CCATAGCCCAGCTCAAGAAG |
| LOC101890093 | Proclotting enzyme | ACAACGACACCAACCACTGA | CGTGCACTGATGATGAGCTT |
| LOC101896914 | Attacin-A | GCAACGGCTAATTTGTGGAC | CCAAGACCACCACCAAAATC |
| LOC101893852 | Sarcotoxin II-1 | TCTGAATGCTGGTGTTACGC | TAACCACTTCGAACCCGAAC |
| LOC101897067 | Diptericin-D | CAGCCACCTCCTTCACAAAT | CCACGGTAATCAGGACGACT |
| LOC101888541 | Protein bicaudal C | AACAGGCCTATCCTCCGTTT | CCCAAGAGGACACCATCACT |
| LOC101894377 | Tenascin | CGTCTGCCACAACGATACAC | CAAACCCATCGAGGCAATAC |
| LOC101900389 | Alpha-amylase A | CCAACACTCGTCCCTTCATT | CCCAAGCAGTACCCCAGTTA |
| LOC101891114 | Meiosis arrest female protein | CTTGTGGGCAACACTGAATG | CAATGGTGGTCTTGTCGATG |
| LOC101894036 | Chorion peroxidase | CTTTTCAGGTGCTGCCTTTC | CTTTTCAGGTGCTGCCTTTC |
| LOC101888782 | Lectin subunit alpha | CTTTTCAGGTGCTGCCTTTC | ATTGGGGTTGCTCTTCACAG |
| LOC101891084 | Vitellogenin-1 | TGGAGAAGATACCCGCTTTG | TGTAGACGGGAACACTGCTG |
| LOC101900923 | Protein will die slowly | GCATTTGGGATACAGCCAGT | CTGCCCGATACAATCCACTT |
| LOC101890236 | Importin subunit alpha-4 | CGTCTGGGCTTTGGGTAATA | CACCAGGATGTTGGTGTCTG |
| LOC101900470 | Cold shock domain-containing protein E1 | AACGGCAACACTTCGGTATC | CACCTGGGTTCTGGTCTCAT |
| gi_558515959 | Armadillo segment polarity protein | CCGGTGATCGTGAAGAGATT | CGTGATGGTGGATGCAATAG |

**Table S2. Annotations of 108 proteins in the MdSGHV genome.** Descriptions of the best BLASTp homologies to the known viral and/or cellular genes are indicated (Bit Score ≥ 40; Expect value of E-6). The signature domains in the protein sequences are indicated in the last column. Amino acid coordinates of the N-terminal signal peptide (SP) sequence and transmembrane domains (TM) are indicated in the last column. Of the 108 MdSGHV proteins, 31 proteins are putative transmembrane proteins as they contained at least one TM. ORFs marked with asterisks (*****) denote 10 of the most abundant MdSGHV structural proteins based on the RNA-Seq reads at 48 h-pi (Compare with **Figure** 2). The 20 MdSGHV ORFs in bold encode proteins that ate homologous to proteins in the two hytrosaviruses infecting the tsetse fly (GpSGHV-Uga and GpSGHV-Eth; Abd-Alla *et al*., 2016), which have been reported to be functional proteins (i.e. contain TATA-box, poly(A) signal sequences and have been confirmed by RNA-Seq and LC-MS/MS).

| **Description of MdSGHV Protein sequences** | | | | **Best BLAST match (Homologies to known viral and/or cellular proteins)** | | | | | **Signature domains/motifs and functional roles** |
| --- | --- | --- | --- | --- | --- | --- | --- | --- | --- |
| **Acc. No.** | **ORF name** | **Sequence description** | **Length [aa]** | **Best match; [Organism]** | **Identity [%]** | **Acc. No.** | **E-Value** | **Score** |  |
| YP_001883329.1 | MdSGHV001 | DNA polymerase | 977 | DNA polymerase-like protein; [GpSGHV-Uga] | 31 | YP_001687027.1 | 3e-137 | 430 | Coiled coils; DNApol-B delta subfamily catalytic domain; Essential for DNA replication |
| YP_001883330.1 | MdSGHV002 | Hypothetical protein | 193 | No Hits Found |  |  |  |  |  |
| YP_001883331.1 | MdSGHV003 | Hypothetical protein | 156 | No Hits Found |  |  |  |  | TM (107-129) |
| **YP_001883332.1** | **MdSGHV004** | **Hypothetical protein** | **131** | **Virion protein SGHV082; [GpSGHV-Uga]** | **27** | **YP_001687030.1** | **9e-11** | **50** |  |
| YP_001883333.1 | MdSGHV005 | Hypothetical protein | 69 | No Hits Found |  |  |  |  |  |
| YP_001883334.1 | MdSGHV006 | Hypothetical protein | 72 | No Hits Found |  |  |  |  | TM (51-71) |
| YP_001883335.1 | MdSGHV007 | Hypothetical protein | 53 | No Hits Found |  |  |  |  |  |
| YP_001883336.1 | MdSGHV008 | Hypothetical protein | 83 | No Hits Found |  |  |  |  |  |
| YP_001883337.1 | MdSGHV009 | Hypothetical protein | 84 | No Hits Found |  |  |  |  | SP (1-25) |
| YP_001883338.1 | MdSGHV0010 | Mitochondrial carrier (MC) protein | 256 | Mitochondrial carrier protein-like protein; [OrNV] | 39 | YP_002321322.1 | 3e-56 | 183 | TM (6-27; 48-67; 87-109; 151-170; 182-203); Important for mitochondrial transport |
| YP_001883339.1 | MdSGHV011 | Dihydrofolate reductase (DHFR) | 170 | Dihydrofolate reductase; [*Aeromicrobium marinum* DSM 15272] | 32 | EFQ82433.1 | 4e-16 | 80.1 | SP (1-19); Essential for thymidilate synthesis and DNA replication |
| **YP_001883340.1** | **MdSGHV012** | **Thymidylate synthase** | **294** | **Thymidylate synthase/pyrimidine hydroxymethylase-like protein [OrNV; [GpSGHV-Uga]** | **54** | **YP_002321317.1** | **1e-109** | **322** | **Pyrimidine nucleobase metabolism** |
| **YP_001883341.1** | **MdSGHV013** | **Hypothetical protein** | **644** | **Nucleocapsid protein SGHV083; [GpSGHV-Uga]** | **27.3** | **YP_001687031.1** | **8e-24** | **99.8** |  |
| YP_001883342.1 | MdSGHV014 | Molybdopterin oxidoreductase (mopB) | 249 | No Hits Found |  |  |  |  | Major outer membrane protein B (MopB) |
| YP_001883343.1 | MdSGHV015 | Hypothetical protein | 299 | No Hits Found |  |  |  |  |  |
| YP_001883344.1 | MdSGHV016***** | Hypothetical protein | 499 | No Hits Found |  |  |  |  | TM (179-200) |
| YP_001883345.1 | MdSGHV017 | Hypothetical protein | 553 | Metalloprotease-like protein; [GpSGHV-Eth] | 24 | AMB48700.1 | 2e-07 | 47.4 | TM (6-23) |
| YP_001883346.1 | MdSGHV018 | Hypothetical protein | 233 | No Hits Found |  |  |  |  |  |
| YP_001883347.1 | MdSGHV019 | Hypothetical protein | 225 | No Hits Found |  |  |  |  |  |
| YP_001883348.1 | MdSGHV020 | Hypothetical protein | 104 | No Hits Found |  |  |  |  | TM (44-66; 78-100) |
| YP_001883349.1 | MdSGHV021 | Hypothetical protein | 644 | Putative serine-rich protein; [*Escovopsis weberi*] | 33.1 | A0A0M9VWC6 | 9e-07 | 149 | Coiled coils |
| YP_001883350.1 | MdSGHV022 | Hypothetical protein | 343 | Tegument protein SGHV093; [GpSGHV-Uga] | 25 | YP_001687041.1 | 3e-37 | 130 |  |
| YP_001883351.1 | MdSGHV023***** | Hypothetical protein | 256 | No Hits Found |  |  |  |  |  |
| YP_001883352.1 | MdSGHV024 | Hypothetical protein | 128 | No Hits Found |  |  |  |  |  |
| **YP_001883353.1** | **MdSGHV025** | **Hypothetical protein** | **376** | **Vesicle-associated membrane-like protein; [GpSGHV-Eth]** | **33** | **AMB48710.1** | **3e-61** | **196** | **TM (162-181; 313-335)** |
| YP_001883354.1 | MdSGHV026 | Hypothetical protein | 60 | No Hits Found |  |  |  |  | SP (1-32) |
| YP_001883355.1 | MdSGHV027 | Hypothetical protein | 169 | No Hits Found |  |  |  |  |  |
| YP_001883356.1 | MdSGHV028***** | Hypothetical protein | 381 | No Hits Found |  |  |  |  |  |
| **YP_001883357.1** | **MdSGHV029** | ***per os* infectivity factor PIF-1** | **644** | **PIF-1; [GpSGHV-Eth]** | **33** | **AMB48716.1** | **1e-100** | **315** | **TM (6-24); A structural protein of baculovirus ODV envelop required for infectivity** |
| **YP_001883358.1** | **MdSGHV030** | **Hypothetical protein** | **692** | **Nucleocapsid protein; [GpSGHV-Eth]** | **24** | **AMB48718.1** | **1e-52** | **187** |  |
| YP_001883359.1 | MdSGHV031 | Hypothetical protein | 66 | No Hits Found |  |  |  |  | Coiled coils; TM (12-34) |
| YP_001883360.1 | MdSGHV032 | Hypothetical protein | 393 | No Hits Found |  |  |  |  | Coiled coils |
| **YP_001883361.1** | **MdSGHV033** | **Vacuolar sorting-associated 4A (AAA+_ATPase)** | **497** | **Cell division protein 48-like protein; [GpSGHV-Uga]** | **33** | **YP_001687055.1** | **8e-49** | **169** | **DEAD-box subfamily ATP-dependent helicases Involved in diverse cellular functions** |
| YP_001883362.1 | MdSGHV034 | Hypothetical protein | 64 | No Hits Found |  |  |  |  | TM (17-39) |
| YP_001883363.1 | MdSGHV035 | Hypothetical protein | 242 | No Hits Found |  |  |  |  | TM (7-29) |
| **YP_001883364.1** | **MdSGHV036** | **Zinc-dependent matrix metalloproteinase 14 (MMP-14)** | **196** | **MP-NASE-like protein; [GpSGHV-Eth]** | **28** | **AMB48725.1** | **2e-20** | **79.3** | **TM (3-21); Vital in morphogenesis, cell fate specification, cell migration, tissue repair, proliferation, and apoptosis** |
| YP_001883365.1 | MdSGHV037 | Small nuclear RNA activating complex, subunit 2, SNAP190 Myb | 294 | Protease; [SuHV-1] | 29 | AJD79504.1 | 7e-04 | 41.6 | SP (1-22); TM (230-256); Prokaryotic membrane lipoprotein lipid attachment site domain; Involved in recognition of TATA box during transcription |
| YP_001883366.1 | MdSGHV038 | Hypothetical protein | 250 | No Hits Found |  |  |  |  | Coiled coils |
| **YP_001883367.1** | **MdSGHV039** | ***per os* infectivity factor P74** | **707** | **P74; [GpSGHV-Eth]** | **40** | **AMB48605.1** | **3e-174** | **509** | **TM (667-684; 690-706); Viral life cycle** |
| YP_001883368.1 | MdSGHV040***** | Hypothetical protein | 608 | No Hits Found |  |  |  |  |  |
| YP_001883369.1 | MdSGHV041 | Hypothetical protein | 441 | No Hits Found |  |  |  |  |  |
| YP_001883370.1 | MdSGHV042 | Hypothetical protein | 627 | No Hits Found |  |  |  |  |  |
| YP_001883371.1 | MdSGHV043 | Hypothetical protein | 369 | No Hits Found |  |  |  |  |  |
| YP_001883372.1 | MdSGHV044 | Hypothetical protein | 311 | No Hits Found |  |  |  |  | SP (1-18) |
| YP_001883373.1 | MdSGHV045 | Hypothetical protein | 380 | No Hits Found |  |  |  |  |  |
| YP_001883374.1 | MdSGHV046 | Lecithin: cholesterol acyltransferase (LCAT)-like protein | 395 | Putative lecithine cholesterol acyltransferase; [GpSGHV-Eth] | 29 | AMB48609.1 | 4e-39 | 137 | TM (118-140; 162-184); Acyl-carrier-protein biosynthesis/lipid metabolisms |
| YP_001883375.1 | MdSGHV047 * | Occlusion derived virus envelope 66 (ODV-e66) | 700 | ODV-E66 protein; [GpSGHV-Eth] | 36 | AMB48608.1 | 2e-55 | 187 | TM (18-39); Has essential role in *per os* infection route |
| YP_001883376.1 | MdSGHV048 | Ring-infected erythrocyte surface antigen | 390 | Ring-infected erythrocyte surface antigen; [*P. reichenowi*] | 47 | XP_012760776.1 | 7e-04 | 45.4 | SP (1-23); A TspB virulence factor-like protein |
| YP_001883377.1 | MdSGHV049 | Ac150-like protein | 167 | AcOrf-150 peptide; [AcMNPV] | 35 | NP_054181.1 | 4e-12 | 62.0 | TM (52-74); Chitin-binding type-2 domain; expressed late during baculovirus infection |
| YP_001883378.1 | MdSGHV050 | M20 Aminoacylase-1-like (ACY1) protein | 404 | Aminoacylase-1-like isoform X1; [*Diuraphis noxia*] | 32 | XP_015377278.1 | 4e-48 | 177 | Obsolete urea cycle intermediate metabolism |
| YP_001883379.1 | MdSGHV051 | Hypothetical protein | 317 | No Hits Found |  |  |  |  |  |
| YP_001883380.1 | MdSGHV052 | Deoxyuridine 5'-triphosphate nucleotidohydrolase (dUTP) | 150 | dUTP-like protein; [*Vigna radiata* var. radiata] | 60 | XP_014505188.1 | 4e-46 | 156 | Central role is maintenance of cellular dUTP levels to prevent dUTP incorporation into DNA |
| YP_001883381.1 | MdSGHV053 | Hypothetical protein | 146 | No Hits Found |  |  |  |  |  |
| YP_001883382.1 | MdSGHV054 | Hypothetical protein | 161 | No Hits Found |  |  |  |  |  |
| **YP_001883383.1** | **MdSGHV055** | **Hypothetical protein** | **416** | **Casein kinase isoform1-D-like protein; [GpSGHV-Eth]** | **26** | **AMB48646.1** | **1e-19** | **84.3** |  |
| YP_001883384.1 | MdSGHV056 | Hypothetical protein | 115 | No Hits Found |  |  |  |  |  |
| YP_001883385.1 | MdSGHV057 | Hypothetical protein | 73 | No Hits Found |  |  |  |  | TM (6-23) |
| YP_001883386.1 | MdSGHV058 | Hypothetical protein | 131 | No Hits Found |  |  |  |  |  |
| YP_001883387.1 | MdSGHV059 | Hypothetical protein | 65 | No Hits Found |  |  |  |  |  |
| YP_001883388.1 | MdSGHV060 | Hypothetical protein | 51 | No Hits Found |  |  |  |  |  |
| YP_001883389.1 | MdSGHV061 | Transposase-like protein | 107 | No Hits Found |  |  |  |  |  |
| YP_001883390.1 | MdSGHV062 | Ribonucleoside-diphosphate reductase subunit M2 (RR2) | 343 | RR2b; [LyxyMNPV] | 75 | YP_003517877.1 | 0.0 | 513 | TM (172-193); Involved in diverse functions e.g. iron regulation, mono-oxygenation, reactive radical production |
| YP_001883391.1 | MdSGHV063 | Hypothetical protein | 90 | No Hits Found |  |  |  |  |  |
| YP_001883392.1 | MdSGHV064 | E3 ubiquitin ligase RNF146 (Iduna) | 475 | No Hits Found |  |  |  |  | Zinc finger RING-type/ ATP-cone domain; Diverse functions e.g. protein-protein interactions, viral replication, signal transduction |
| YP_001883393.1 | MdSGHV065 | Class I ribonucleotide reductase-diphosphate reductase (RR1) | 785 | RR1; [SltMNPV] | 61 | ADE80903.1 | 0.0 | 1019 | RR1-large subunit domain; Provides precursors necessary for DNA synthesis |
| YP_001883394.1 | MdSGHV066 | Na^+^-dependent nucleoside transporter, co-transporter II (CNT2) | 579 | Sodium/nucleoside co-transporter 2; [*Drosophila bipectinata*] | 26 | XP_017093821.1 | 5e-38 | 158 | TM (54-75; 84-99; 125-147; 150-169; 198-220; 230-252; 269-291; 307-329; 333-355; 424-446; 459-481; 553-575); Nucleoside transmembrane transport |
| YP_001883395.1 | MdSGHV067 | Hypothetical protein | 162 | No Hits Found |  |  |  |  |  |
| YP_001883396.1 | MdSGHV068 | Hypothetical protein | 132 | No Hits Found |  |  |  |  | TM (57-79; 86-108) |
| YP_001883397.1 | MdSGHV069 | Thymidine kinase (TK) | 203 | Putative deoxynucleoside kinase protein; [ATCV-1] | 32 | AGE57698.1 | 2e-16 | 76.6 | Diverse roles in nucleotide metabolism, coenzyme biosynthesis and sugar/sulphate metabolism |
| **YP_001883398.1** | **MdSGHV070** | **Hypothetical protein** | **967** | **LEF8-like protein; [GpSGHV-Eth]** | **35** | **AMB48645.1** | **2e-179** | **538** | **TM (469-491)** |
| **YP_001883399.1** | **MdSGHV071 *** | **Hypothetical protein** | **333** | **No Hits Found** |  |  |  |  |  |
| YP_001883400.1 | MdSGHV072 | Metallophosphoesterase (MPPE)-like protein | 136 | No Hits Found |  |  |  |  | TM (113-134); Belongs to the *Enterobacter aerogenes* GpdQ and related proteins with diverse functions |
| **YP_001883401.1** | **MdSGHV073** | **Hypothetical protein** | **390** | **Nucleocapsid protein; [GpSGHV-Eth]** | **34** | **AMB48649.1** | **7e-10** | **53.3** |  |
| **YP_001883402.1** | **MdSGHV074** | **Hypothetical protein** | **698** | **LEF-9-like protein; GpSGHV-Eth]** | **26** | **AMB48638.1** | **6e-59** | **196** | **SP (1-15)** |
| YP_001883403.1 | MdSGHV075 | Hypothetical protein | 108 | No Hits Found |  |  |  |  |  |
| YP_001883404.1 | MdSGHV076 | Hypothetical protein | 382 | Polyhedrin/Granulin (polh/gran) protein; [OrNV] | 38 | YP_002321327.1 | 2e-80 | 249 |  |
| YP_001883405.1 | MdSGHV077 | Hypothetical protein | 298 | No Hits Found |  |  |  |  |  |
| YP_001883406.1 | MdSGHV078 | Death-associated inhibitor of apoptosis 2-like (IAP) | 142 | ORF MSV248 putative IAP; [MsEPV] | 34 | NP_048319.1 | 1e-26 | 94 | BIR-and RING-finger-containing E3 ubiquitin ligase domain-containing protein (BIRP) |
| YP_001883407.1 | MdSGHV079 | Hypothetical protein | 122 | No Hits Found |  |  |  |  | TM (4-26) |
| YP_001883408.1 | MdSGHV080 | Hypothetical protein | 94 | No Hits Found |  |  |  |  |  |
| YP_001883409.1 | MdSGHV081 | Hypothetical protein | 94 | No Hits Found |  |  |  |  |  |
| YP_001883410.1 | MdSGHV082 | RCI site-specific recombinase-like | 359 | No Hits Found |  |  |  |  | Shufflon-specific DNA recombinase Rci and Bacteriophage Hp1_like integrase |
| **YP_001883411.1** | **MdSGHV083** | **Hypothetical protein** | **1780** | **LEF3-like protein; [GpSGHV-Eth]** | **26** | **AMB48650.1** | **3e-94** | **332** | **TM (151-171)** |
| **YP_001883412.1** | **MdSGHV084** | **Hypothetical protein** | **509** | **Glutathione-S-transferase-like protein; [GpSGHV-Eth]** | **23** | **AMB48652.1** | **2e-35** | **132** |  |
| YP_001883413.1 | MdSGHV085***** | Hypothetical protein | 403 | No Hits Found |  |  |  |  | TM (148-165) |
| YP_001883414.1 | MdSGHV086***** | Hypothetical protein | 381 | No Hits Found |  |  |  |  | Contains nuclear lamina binding domain; putative viral matrix protein |
| **YP_001883415.1** | **MdSGHV087** | **Hypothetical protein** | **544** | **LEF4-like protein; [GpSGHV-Eth]** | **23** | **AMB48656.1** | **4e-12** | **62** |  |
| YP_001883416.1 | MdSGHV088 | Hypothetical protein | 179 | No Hits Found |  |  |  |  | TM (28-50; 72-94) |
| **YP_001883417.1** | **MdSGHV089** | ***per os* infectivity factor PIF-2** | **379** | **PIF-2; [GpSGHV-Uga]** | **38** | **AMB48658.1** | **5e-82** | **249** | **TM (9-31); Viral life cycle (oral infection)** |
| YP_001883418.1 | MdSGHV090 | Hypothetical protein | 672 | Capsid protein 3; [GpSGHV-Eth] | 25 | AMB48682.1 | 1e-13 | 67.4 | TM (655-672) |
| YP_001883419.1 | MdSGHV091 | Hypothetical protein | 423 | No Hits Found |  |  |  |  |  |
| YP_001883420.1 | MdSGHV092 | Hypothetical protein | 73 | No Hits Found |  |  |  |  | SP (1-29 ); TM (5-26) |
| YP_001883421.1 | MdSGHV093 | Hypothetical protein | 283 | No Hits Found |  |  |  |  | TM (18-40) |
| YP_001883422.1 | MdSGHV094 | Transmembrane protein Meckelin (TMEM67)-like (MKS3) | 111 | No Hits Found |  |  |  |  | TM (68-90) |
| YP_001883423.1 | MdSGHV095 | Hypothetical protein | 140 | No Hits Found |  |  |  |  |  |
| YP_001883424.1 | MdSGHV096***** | A major envelop protein | 1473 | No Hits Found |  |  |  |  |  |
| YP_001883425.1 | MdSGHV097***** | Hypothetical protein | 482 | No Hits Found |  |  |  |  |  |
| YP_001883426.1 | MdSGHV098 | Hypothetical protein | 404 | No Hits Found |  |  |  |  | TM (23-45; 66-88; 94-111; 117-136; 140-162; 164-186; 224-246; 262-284; 291-313; 320-342; 382-404) |
| YP_001883427.1 | MdSGHV099 | Hypothetical protein | 612 | No Hits Found |  |  |  |  |  |
| YP_001883428.1 | MdSGHV100 | Hypothetical protein | 455 | Nucleocapsid protein; [GpSGHV-Eth] | 24 | AMB48672.1 | 4e-19 | 83.2 |  |
| YP_001883429.1 | MdSGHV101 | Hypothetical protein | 198 | No Hits Found |  |  |  |  |  |
| **YP_001883430.1** | **MdSGHV102** | **Hypothetical protein** | **257** | **FAD dependent sulfhydryl oxidase-like protein; [GpSGHV-Uga]** | **37** | **AMB48683.1** | **6e-19** | **77.8** | **ERV/ALR sulfhydryl oxidase domain; involved in redox reactions** |
| YP_001883431.1 | MdSGHV103 | Hypothetical protein | 67 | No Hits Found |  |  |  |  |  |
| YP_001883432.1 | MdSGHV104 | DNA helicase-like protein | 770 | Helicase-2-like protein; [GpSGHV-Eth] | 32 | AMB48685.1 | 3e-67 | 230 |  |
| YP_001883433.1 | MdSGHV105 | Hypothetical protein | 241 | No Hits Found |  |  |  |  | SP (1-34) |
| YP_001883434.1 | MdSGHV106 | *per os* infectivity factor PIF-3 | 242 | PIF-3; [GpSGHV-Uga] | 32 | YP_001687024.1 | 8e-12 | 56.6 | TM (38-60); Viral life cycle (oral infection) |
| **YP_001883435.1** | **MdSGHV107** | **Hypothetical protein** | **1125** | **ATP-binding cassette transporter; [GpSGHV-Eth]** | **28** | **AMB48688.1** | **2e-12** | **65.1** |  |
| **YP_001883436.1** | **MdSGHV108** | **Ac81-like protein** | **205** | **Ac81-like protein; [GpSGHV-Uga** | **35** | **YP_001687026.1** | **6e-39** | **128** | **TM (134-156; 163-184); interacts with Actin 3 in cell cytoplasm during baculovirus infection** |

**Table S3. Gene Ontologies (GO) of downregulated host proteins in viremic houseflies.** Annotations and description of the GO terms associated with the top 50 genes (padj values of ≤ 0.01) in the housefly that were negatively regulated by MdSGHV infection at 48pi. For the annotations, the translated protein sequences were initially blasted using BLASTpBlast2GO (Conesa *et al*., 2005). Further annotations were performed at the nr-NCBI database (Bitscore ≥ 80; E-value ≤ 1.0E-6) on *Drosophilidae* database. A total of 14 uncharacterized housefly sequences (marked with asterisks; *****) yielding uncharacterized *Drosophila* homologs were assigned the closest characterized dipteran homologs available at the nr-NCBI database.

| **Description of MdSGHV genes** | | | **Descriptions of *Drosophila* homologs** | | | | | **GO term description** |
| --- | --- | --- | --- | --- | --- | --- | --- | --- |
| **Gene identity** | **Log2FC** | **Sequence name** | **Sequence name; [species name]** | **Identity [%]** | **Acc. No.** | **E-Value** | **Score** |  |
| gi\|557782804\|ref\|XP_005191012.1\| | -10.04 | Vitellogenin-1-like | Vitellogenin-1; [*D. miranda*] | 52 | XP_017156634.1 | 2e-149 | 434 | CC: lipid particle; BP: sex differentiation; BP: response to bacterium; BP: vitellogenesis; CC: P granule; MF: structural molecule activity; BP: regulation of embryonic development; BP: neurogenesis; CC: extracellular region; CC: microtubule associated complex; MF: hydrolase activity |
| gi\|557769075\|ref\|XP_005184212.1\| | -10.02 | Vitellogenin-1-like | Vitellogenin-1; [*D. miranda*] | 47 | XP_017156634.1 | 1e-135 | 398 | BP: metabolic process; CC: extracellular region; MF: carboxylic ester hydrolase activity; MF: hydrolase activity |
| gi\|557776288\|ref\|XP_005187787.1\| | -9.93 | Lectin subunit alpha-like | Lectin subunit alpha-like; [*D. miranda*] | 30 | XP_017140224.1 | 7e-11 | 63.9 | MF: carbohydrate binding |
| gi\|557769073\|ref\|XP_005184211.1\| | -9.48 | Vitellogenin-3-like | Vitellogenin-3; [*D. rhopaloa*] | 53 | XP_016974670.1 | 6e-156 | 449 | BP: metabolic process; CC: extracellular region; MF: carboxylic ester hydrolase activity |
| gi\|557782800\|ref\|XP_005191010.1\| | -8.80 | DyrK-mbk-2-like | DyrK3; [*D. miranda*] | 86 | XP_017155478.1 | 0.0 | 835 | MF: ATP binding; MF: protein kinase activity; BP: protein phosphorylation |
| gi\|755877390\|ref\|XP_005184354.2\|***** | -8.02 | Uncharacterized protein LOC101898197 | Hypothetical protein FF38_06183; [*L. cuprina*] | 56 | KNC27920.1 | 0.0 | 1035 | CC: lipid particle; MF: hydrolase activity |
| gi\|557765600\|ref\|XP_005182499.1\| | -7.75 | Uncharacterized protein LOC101891285 | Tsal1 protein precursor; [*G. m. morsitans*] | 34 | AAF82097.1 | 2e-80 | 247 | MF: nucleic acid binding; MF: metal ion binding; BP: metabolic process; MF: hydrolase activity |
| gi\|755851053\|ref\|XP_011291688.1\| | -7.74 | GlcDH-(FAD, quinone) | GlcDH-(FAD, quinone); [*D. suzukii*] | 68 | XP_016923969.1 | 0.0 | 538 | MF: flavin adenine dinucleotide binding; BP: pentose-phosphate shunt; CC: integral component of membrane; BP: oxidation-reduction process; BP: ecdysteroid metabolic process; MF: glucose dehydrogenase activity; CC: chorion |
| gi\|557751295\|ref\|XP_005175404.1\| | -7.63 | GlcDH-(FAD, quinone)-like | GH12758; [*D. grimshawi*] | 41 | XP_001991629.1 | 2e-136 | 416 | MF: oxidoreductase activity; BP: mesoderm development |
| gi\|557765598\|ref\|XP_005182498.1\|***** | -7.58 | Uncharacterized protein LOC101891113 | Tsal2-A; [*G. m. morsitans*] | 35 | ABN58709.1 | 9e-75 | 231 | MF: binding |
| gi\|557760194\|ref\|XP_005179820.1\| | -7.24 | Probable phospholipid-transporting ATPase IF | Phospholipid-transporting ATPase IF; [*D. eugracilis*] | 59 | XP_017063690.1 | 0.0 | 791 | MF: nucleotide binding; MF: metal ion binding; CC: membrane; BP: phospholipid transport; MF: hydrolase activity |
| gi\|822092655\|ref\|NP_001295965.1\| | -6.46 | Vitellogenin-1-like precursor | Vitellogenin-3; [*D. rhopaloa*] | 51 | XP_016974670.1 | 2e-145 | 422 | BP: metabolic process; CC: extracellular region; MF: carboxylic ester hydrolase activity |
| gi\|755893207\|ref\|XP_011295171.1\| | -6.22 | Serine protease nudel (Ndl) | Ndl; [*D. miranda*] | 48 | XP_017135942.1 | 0.0 | 2283 | BP: Toll signaling pathway; BP: eggshell chorion assembly; CC: membrane; MF: serine-type peptidase activity; BP: protein processing; BP: egg activation; BP: maternal specification of dorsal/ventral axis, oocyte, soma encoded |
| gi\|557776277\|ref\|XP_005187782.1\| | -6.12 | Uncharacterized transmembrane DDB_G0289901-like | Vitelline membrane protein-26Ab; [*D. suzukii*] | 65 | XP_016935818.1 | 2e-17 | 82 |  |
| gi\|557771819\|ref\|XP_005185568.1\|***** | -6.07 | TFIID | TFIID subunit 3; [*S. calcitrans*] | 61 | XP_013107927.1 | 0.0 | 1743 | MF: binding |
| gi\|557754326\|ref\|XP_005176907.1\| | -5.80 | Larval serum protein 2-like (hexamerin) | Uncharacterized protein Dwil_GK19679; [*D. willistoni*] | 61 | XP_002062868.1 | 0.0 | 897 |  |
| gi\|557755346\|ref\|XP_005177413.1\|***** | -5.55 | Uncharacterized protein LOC101899278 | Uncharacterized protein LOC108044774; [*D. rhopaloa*] | 38 | XP_016979403.1 | 7e-38 | 132 |  |
| gi\|755894271\|ref\|XP_011295332.1\| | -5.55 | Vitellogenin receptor | Putative vitellogenin receptor; [*D. arizonae*] | 54 | XP_017872549.1 | 0.0 | 2132 | MF: calcium ion binding; CC: integral component of membrane |
| gi\|755876995\|ref\|XP_011292789.1\| | -5.30 | E3 ubiquitin- ligase UBR1 | E3 ubiquitin-protein ligase UBR1; [*D. biarmipes*] | 57 | XP_016962740.1 | 0.0 | 2083 | MF: zinc ion binding; BP: protein catabolic process; MF: ligase activity |
| gi\|557754967\|ref\|XP_005177226.1\|***** | -5.27 | Uncharacterized protein LOC101897949 | Alkaline nuclease; [*An. darlingi*] | 39 | ETN61460.1 | 6e-99 | 304 | MF: nucleic acid binding; MF: metal ion binding; BP: metabolic process; MF: hydrolase activity |
| gi\|557758281\|ref\|XP_005178869.1\| | -5.06 | F-box only 33 | F-box only protein 33; [*D. ficusphila*] | 59 | XP_002069129.1 | 0.0 | 574 |  |
| gi\|557781660\|ref\|XP_005190448.1\| | -4.98 | Vitellogenin-2-like | Yolk protein 2a; [*D. melanogaster*] | 50 | NP_511102.3 | 1e-155 | 451 | CC: lipid particle; BP: sex differentiation; CC: ACF complex; BP: oogenesis; BP: metabolic process; BP: neurogenesis; CC: extracellular region; CC: microtubule associated complex; MF: carboxylic ester hydrolase activity |
| gi\|557756384\|ref\|XP_005177929.1\| | -4.97 | Kinesin-like protein KIF16B | Kinesin-like protein at 98A; [*D. melanogaster*] | 71 | NP_001247339.1 | 0.0 | 1739 | MF: ATP binding; MF: microtubule motor activity; CC: endosome; CC: tubulin complex; MF: phosphatidylinositol phosphate binding; BP: metabolic process; BP: early endosome to late endosome transport; CC: kinesin complex; BP: microtubule-based movement; CC: microtubule; MF: microtubule binding |
| gi\|557771252\|ref\|XP_005185288.1\| | -4.95 | Alpha-amylase A | Alpha-amylase B-like; [*D. busckii*] | 59 | XP_017839438.1 | 0.0 | 573 | MF: alpha-amylase activity; MF: cation binding; BP: starch metabolic process; BP: sucrose metabolic process |
| gi\|755893977\|ref\|XP_011295288.1\| | -4.92 | Uncharacterized protein LOC105262316 | Uncharacterized protein LOC108036880; [*D. biarmipes*] | 56 | XP_016968744.1 | 2e-40 | 141 | MF: DNA binding; MF: methyltransferase activity; MF: transferase activity; BP: methylation |
| gi\|755886251\|ref\|XP_005187568.2\|***** | -4.85 | Uncharacterized protein LOC101895154 | Uncharacterized protein LOC108114207; [*D. eugracilis*] | 34 | XP_017080516.1 | 6e-117 | 363 | CC: membrane; CC: integral component of membrane; BP: sensory perception of pain |
| gi\|755888261\|ref\|XP_005188217.2\|***** | -4.79 | Uncharacterized protein LOC101887975 | Protein takeout-like; [*S. calcitrans*] | 53 | XP_013107684.1 | 7e-88 | 275 |  |
| gi\|557757763\|ref\|XP_005178613.1\| | -4.79 | Integrator complex subunit 10 | Integrator complex subunit 10; [*D. rhopaloa*] | 56 | XP_016979162.1 | 0.0 | 719 | BP: snRNA processing; CC: integrator complex |
| gi\|755880418\|ref\|XP_005185446.2\| | -4.72 | Alpha-amylase A-like | Alpha-amylase A-like; [*D. eugracilis*] | 75 | XP_017078808.1 | 0.0 | 798 | MF: alpha-amylase activity; MF: metal ion binding; BP: starch metabolic process; BP: sucrose metabolic process |
| gi\|557762682\|ref\|XP_005181055.1\| | -4.71 | Inner centromere protein-like | Inner centromere protein; [*D. navojoa*] | 55 | XP_017962084.1 | 4e-34 | 130 | CC: nucleus; BP: meiotic chromosome segregation; CC: chromosome, centromeric region |
| gi\|755872279\|ref\|XP_011292102.1\| | -4.66 | Meiosis arrest female 1 | Meiosis arrest female protein 1; [*D. miranda*] | 63 | XP_017152641.1 | 0.0 | 1407 | MF: nucleotide binding; CC: peroxisome; BP: regulation of gene expression |
| gi\|557760463\|ref\|XP_005179952.1\| | -4.66 | D-beta-hydroxybutyrate, mitochondrial | D-beta-hydroxybutyrate dehydrogenase, mitochondrial; [*D. eugracilis*] | 61 | XP_017076389.1 | 1e-151 | 432 | MF: 3-hydroxybutyrate dehydrogenase activity; BP: cellular ketone body metabolic process; BP: ecdysone biosynthetic process |
| gi\|557776288\|ref\|XP_005187787.1\| | -4.57 | Lectin subunit alpha-like | Lectin subunit alpha-like; [*D. miranda*] | 30 | XP_017140224.1 | 7e-11 | 63.9 | MF: carbohydrate binding |
| gi\|557777779\|ref\|XP_005188523.1\|***** | -4.54 | Uncharacterized protein LOC101893663 | Uncharacterized protein Dana_GF24445; [*D. ananassae*] | 60 | XP_001956707.2 | 2e-96 | 303 |  |
| gi\|557780756\|ref\|XP_005189999.1\|***** | -4.52 | Uncharacterized protein LOC101896665 | Uncharacterized protein LOC108609936; [*D. arizonae*] | 45 | XP_017857212.1 | 1e-68 | 216 | MF: lipid binding |
| gi\|557759511\|ref\|XP_005179480.1\| | -4.51 | Homeobox protein araucan | Homeobox protein caupolican; [*D. busckii*] | 71 | XP_017843929.1 | 0.0 | 729 | CC: nucleus; BP: regulation of transcription, DNA-templated; MF: sequence-specific DNA binding |
| gi\|755875228\|ref\|XP_011292538.1\|***** | -4.50 | Uncharacterized protein LOC101900380 - isoform X1 | Zonadhesin-like; [*S. calcitrans*] | 54 | XP_013108514.1 | 0.0 | 2257 | MF: chitin binding; MF: structural constituent of peritrophic membrane; BP: chitin metabolic process; CC: extracellular region |
| gi\|557751929\|ref\|XP_005175717.1\| | -4.47 | Uncharacterized protein LOC101890573 | Gag-pol protein; [*D. ananassae*] | 37 | ABP48078.1 | 9e-135 | 438 | MF: nucleic acid binding; BP: DNA integration |
| gi\|755876551\|ref\|XP_011292728.1\| | -4.42 | Chorion peroxidase | Chorion peroxidase; [*D. takahashii*] | 62 | XP_017014543.1 | 0.0 | 1095 | MF: peroxidase activity; MF: prostaglandin-endoperoxide synthase activity; BP: response to oxidative stress; BP: eggshell chorion assembly; BP: leukotriene metabolic process; BP: obsolete peroxidase reaction; BP: prostaglandin biosynthetic process; BP: cellular oxidant detoxification; BP: oxidation-reduction process; CC: microtubule associated complex; BP: hydrogen peroxide metabolic process; MF: heme binding |
| gi\|755886141\|ref\|XP_011294153.1\| | -4.40 | Uncharacterized protein LOC101900801 | Discoidin domain-containing receptor 2; [*D. kikkawai*] | 69 | XP_017033755.1 | 3e-77 | 269 | CC: integral component of membrane; MF: protein kinase activity; BP: protein phosphorylation |
| gi\|557759481\|ref\|XP_005179465.1\| | -4.35 | Acyl-COA-binding protein-like | Acyl-CoA-binding protein homolog; [*D. busckii*] | 77 | XP_017842368.1 | 6e-42 | 135 | MF: fatty-acyl-CoA binding |
| gi\|557773073\|ref\|XP_005186190.1\| | -4.34 | Serine/threonine- kinase Aurora-2 | Aurora kinase C; [*D. kikkawai*] | 68 | XP_017037810.1 | 0.0 | 516 | BP: pole cell formation; BP: establishment of spindle orientation; BP: mitotic centrosome separation; BP: serine family amino acid metabolic process; MF: protein serine/threonine kinase activity; MF: ATP binding; CC: centrosome; BP: regulation of cellular protein localization; MF: protein serine/threonine/tyrosine kinase activity; CC: cytoplasm; BP: asymmetric protein localization involved in cell fate determination; BP: centrosome duplication; BP: symmetric cell division; BP: peptidyl-serine phosphorylation; BP: regulation of neurogenesis |
| gi\|557775540\|ref\|XP_005187416.1\| | -4.26 | Epithelial discoidin domain-containing receptor 1-like | Uncharacterized protein Dere_GG10403; [*D. erecta*] | 62 | XP_001969997.2 | 7e-52 | 174 | CC: membrane; MF: protein kinase activity; BP: protein phosphorylation |
| gi\|755893538\|ref\|XP_005190193.2\| | -4.26 | Protein bicaudal C-like | Protein bicaudal C; [*D. biarmipes*] | 78 | XP_016962167.1 | 0.0 | 580 | BP: negative regulation of oskar mRNA translation; CC: integral component of plasma membrane; MF: protein binding; BP: ovarian follicle cell migration; MF: mRNA binding; BP: mitotic nuclear division; BP: microtubule cytoskeleton organization; BP: actin cytoskeleton organization |
| gi\|755870064\|ref\|XP_011291776.1\| | -4.21 | Cell division cycle protein 20 homolog | Uncharacterized protein Dmoj_GI17076; [*D. mojavensis*] | 78 | XP_002001863.1 | 0.0 | 853 | CC: anaphase-promoting complex; BP: regulation of exit from mitosis; BP: negative regulation of cyclin-dependent protein serine/threonine kinase by cyclin degradation; BP: regulation of proteolysis; BP: negative regulation of necrotic cell death; BP: female meiosis II; BP: mitotic nuclear division; BP: female meiosis I; BP: neurogenesis; CC: kinetochore; BP: neuronal stem cell population maintenance; BP: cell division; CC: centrosome; MF: ubiquitin-protein transferase activator activity; CC: cytoplasm; BP: germ-line stem cell population maintenance; CC: spindle; BP: phagocytosis; BP: anaphase-promoting complex-dependent catabolic process; MF: anaphase-promoting complex binding |
| gi\|755891145\|ref\|XP_011294885.1\| | -4.21 | Venom allergen 5-like | Venom allergen 3-like; [*D. kikkawai*] | 38 | XP_017017967.1 | 1e-46 | 163 | MF: catalytic activity; BP: metabolic process |
| gi\|755883458\|ref\|XP_005186532.2\|***** | -4.18 | Uncharacterized protein LOC101888900 | Beaten path Ic; [*D. melanogaster*] | 60 | NP_523580.1 | 6e-34 | 140 | CC: integral component of plasma membrane; BP: heterophilic cell-cell adhesion via plasma membrane cell adhesion molecules; BP: axon choice point recognition; CC: extracellular region; BP: defasciculation of motor neuron axon |
| gi\|557782818\|ref\|XP_005191019.1\|***** | -4.16 | Uncharacterized protein LOC101892465 | Peritrophin-15 precursor; [*C. bezziana*] | 60 | AAK01057.1 | 1e-33 | 116 | MF: binding |
| gi\|557770791\|ref\|XP_005185058.1\| | -4.14 | Importin subunit alpha (pendulin) | Importin subunit alpha; [*D. kikkawai*] | 84 | XP_017032683.1 | 0.0 | 919 | BP: cell proliferation; BP: sperm individualization; MF: protein transmembrane transporter activity; BP: female germline ring canal formation; BP: gravitaxis; BP: protein import into nucleus, docking; CC: microtubule associated complex; CC: nuclear pore; BP: cytoplasmic transport, nurse cell to oocyte; CC: lipid particle; CC: cytoplasm; BP: centrosome duplication; BP: lymph gland development; BP: NLS-bearing protein import into nucleus; CC: actin cytoskeleton; BP: sensory perception of pain |
| gi\|755882320\|ref\|XP_005186100.2\|***** | -4.13 | Uncharacterized protein LOC101900797 | MKRN2 opposite strand protein; [*D. busckii*] | 68 | XP_017851727.1 | 4e-88 | 276 |  |

**Table S4. Gene Ontologies (GO) of upregulated host proteins in viremic houseflies.** Top fifty genes having a padj value of ≥0.01 positively regulated by MdSGHV infection at 48pi. Gene information derived from annotation to *M. domestica or Drosophila* databases. For the annotations, the translated protein sequences were initially blasted using BLASTpBlast2GO (Conesa *et al*., 2005). Further annotations were performed at the nr-NCBI database (Bitscore ≥ 80; E-value ≤ 1.0E-6) on *Drosophilidae* database. The seven uncharacterized housefly sequences (marked with asterisks; *****) yielding uncharacterized *Drosophila* homologs were assigned the closest characterized dipteran homologs available at the nr-NCBI database. Five sequences remained without any significant hits to characterized proteins in the databases.

| **Description of MdSGHV genes** | | | **Descriptions of *Drosophila* homologs** | | | | | **GO Names** |
| --- | --- | --- | --- | --- | --- | --- | --- | --- |
| **Gene identity** | **Log2FC** | **Sequence name** | **Sequence name; [species name]** | **Identity [%]** | **Acc. No.** | **E-Value** | **Score** |  |
| gi\|557775863\|ref\|XP_005187576\| | 9.13 | Diptericin-D-like | Diptericin-A; [*D. suzukii*] | 62 | XP_016940553.1 | 1e-22 | 88.2 | CC: extracellular region |
| gi\|557759978\|ref\|XP_005179713\| | 8.16 | Cecropin-A2-like | Cecropin-A2; [*D. suzukii*] | 81 | XP_016938597.1 | 2e-31 | 107 | CC: extracellular space; BP: defense response to Gram-negative bacterium; BP: innate immune response; BP: antibacterial humoral response; BP: salivary gland cell autophagic cell death; BP: defense response to fungus; BP: defense response to Gram-positive bacterium |
| gi\|755878000\|ref\|XP_011292934\| | 7.49 | Heparan sulfate 2-O-sulfotransferase pipe-like | Heparan sulfate 2-O-sulfotransferase pipe; [*D. suzukii*] | 72 | XP_016944242.1 | 6e-172 | 489 | BP: Toll signaling pathway; MF: chondroitin sulfotransferase activity; BP: regulation of multicellular organism growth; MF: protein homodimerization activity; BP: imaginal disc-derived wing morphogenesis; MF: heparan sulfate 2-O-sulfotransferase activity; CC: Golgi apparatus; MF: sequence-specific DNA binding; CC: chromosome; BP: chromatin silencing; BP: pole plasm assembly; BP: olfactory behavior; BP: maternal specification of dorsal/ventral axis, oocyte, soma encoded; MF: POZ domain binding; BP: regulation of chromatin silencing; CC: PcG protein complex; CC: integral component of membrane; BP: protein processing |
| gi\|755875984\|ref\|XP_011292636\| | 7.34 | Prolyl 4-hydroxylase subunit alpha-2-like | Prolyl-4-hydroxylase-alpha SG1; [*D. melanogaster*] | 52 | NP_733376.1 | 6e-105 | 330 | MF: dioxygenase activity; MF: oxidoreductase activity, acting on paired donors, with incorporation or reduction of molecular oxygen; BP: peptidyl-proline hydroxylation; CC: procollagen-proline 4-dioxygenase complex; BP: single-organism metabolic process; BP: salivary gland morphogenesis |
| gi\|755865113\|ref\|XP_005180079\| | 6.94 | Sarcotoxin-2A-like | Sarcotoxin II-1-like; [*S. calcitrans*] | 65 | XP_013106587.1 | 2e-102 | 310 | CC: extracellular region |
| gi\|557759980\|ref\|XP_005179714\| | 6.76 | Sarcotoxin-1C-like | Sarcotoxin-1C-like; [*S. calcitrans*] | 79 | XP_013106068.1 | 2e-29 | 103 | BP: innate immune response; CC: extracellular region; BP: defense response to bacterium |
| gi\|557785303\|ref\|XP_005192252\| | 6.65 | Uncharacterized protein LOC101899042 | Gag-pol polyprotein precursor; [*D. melanogaster*] | 34 | CAD32253.1 | 0.0 | 650 |  |
| gi\|557757569\|ref\|XP_005178516\| | 6.53 | Attacin-A-like | Attacin-A; [*D. busckii*] | 41 | XP_017846277.1 | 1e-34 | 124 | BP: defense response to Gram-negative bacterium; BP: antibacterial humoral response; CC: extracellular region |
| gi\|822092426\|ref\|NP_001295990\| | 6.18 | Attacin-A-like precursor | Attacin-A; [*D. ficusphila*] | 46 | XP_017039201.1 | 2e-46 | 156 | BP: defense response to Gram-negative bacterium; BP: antibacterial humoral response; CC: extracellular region |
| gi\|557757637\|ref\|XP_005178550\| | 5.79 | Attacin-A-like | Attacin-A; [*D. busckii*] | 43 | XP_017846277.1 | 2e-36 | 128 | BP: defense response to Gram-negative bacterium; BP: antibacterial humoral response; CC: extracellular region |
| gi\|557780072\|ref\|XP_005189659\| | 5.66 | Elongation of very long chain fatty acids protein AAEL008004-like | Elongation of very long chain fatty acids protein AAEL008004; [*D. elegans*] | 41 | XP_017114536.1 | 5e-66 | 210 | BP: lipid metabolic process; CC: membrane; CC: integral component of membrane; MF: transferase activity; BP: fatty acid biosynthetic process; BP: fatty acid metabolic process |
| gi\|755899809\|ref\|XP_005191037\| | 5.53 | Nuclear factor NF-kappa-B p110 subunit-like (Relish) | Nuclear factor NF-kappa-B p110 subunit; [*D. ficusphila*] | 23 | XP_017058414.1 | 1e-20 | 101 | MF: DNA binding; CC: nucleus; MF: transcription factor activity, sequence-specific DNA binding; CC: cytoplasm; BP: regulation of transcription, DNA-templated |
| gi\|557750739\|ref\|XP_005175131\| | 5.48 | Uncharacterized protein LOC101893332 | Probable RNA-binding protein 46; [*D. rhopaloa*] | 41 | XP_016969076.1 | 2e-37 | 135 | MF: nucleotide binding; MF: nucleic acid binding |
| gi\|557766434\|ref\|XP_005182912\| | 5.43 | Low density lipoprotein receptor adapter protein 1-A-like | Low density lipoprotein receptor adapter protein 1-B-like; [*S. calcitrans*] | 80 | XP_013109812.1 | 5e-173 | 484 |  |
| gi\|755881033\|ref\|XP_005185664\| | 5.39 | Maltase A1-like | Maltase A1; [*D. ficusphila*] | 72 | XP_017044412.1 | 0.0 | 865 | MF: alpha-1,4-glucosidase activity; CC: glucosidase II complex; MF: cation binding; BP: galactose metabolic process; BP: starch metabolic process; BP: sucrose metabolic process |
| gi\|755852711\|ref\|XP_011294328\|***** | 5.39 | Uncharacterized protein LOC101894897 | No Significant Hit found |  |  |  |  | MF: nucleic acid binding; MF: metal ion binding; BP: metabolic process; CC: membrane; CC: integral component of membrane; MF: hydrolase activity |
| gi\|557767551\|ref\|XP_005183464\|***** | 5.25 | Uncharacterized protein LOC101894268 | No Significant Hit found |  |  |  |  | BP: metabolic process; BP: phosphorylation; MF: transferase activity; MF: kinase activity |
| gi\|755885629\|ref\|XP_011294086\| | 5.17 | Protein FAM92A1-like | Protein FAM92A1 isoform X1; [*D. miranda*] | 51 | XP_017153012.1 | 7e-124 | 370 | CC: nucleus; CC: cytoplasm; BP: negative regulation of protein secretion; BP: signal transduction; MF: signal transducer activity |
| gi\|755851764\|ref\|XP_005175670\| | 5.17 | Tryptophan--tRNA ligase, cytoplasmic-like | Tryptophan--tRNA ligase, cytoplasmic; [*D. arizonae*] | 77 | XP_017856386.1 | 0.0 | 659 | MF: ATP binding; CC: cytoplasm; BP: tryptophanyl-tRNA aminoacylation; BP: tryptophan metabolic process; MF: tryptophan-tRNA ligase activity; BP: dendrite morphogenesis |
| gi\|755849779\|ref\|XP_005174996\| | 5.16 | Protein couch potato-like | Protein couch potato; [*S. calcitrans*] | 65 | XP_013101475.1 | 3e-32 | 134 | MF: nucleotide binding; MF: nucleic acid binding |
| gi\|755904621\|ref\|XP_011295928\| | 5.16 | Alpha-*N*-acetylgalactosaminidase | Alpha-*N*-acetylgalactosaminidase; [*D. arizonae*] | 81 | XP_017858007.1 | 4e-164 | 464 | BP: carbohydrate metabolic process; CC: integral component of membrane; MF: hydrolase activity, hydrolyzing O-glycosyl compounds |
| gi\|557775861\|ref\|XP_005187575\| | 5.15 | Diptericin-D-like | Diptericin-A; [*D. takahashii*] | 46 | XP_017014401.1 | 2e-22 | 87.4 | CC: membrane; CC: extracellular region |
| gi\|755865324\|ref\|XP_011291120\| | 5.10 | Tyrosine-protein kinase Fps85D | Tyrosine-protein kinase Fer isoform X1; [Drosophila arizonae] | 85 | XP_017863571.1 | 0.0 | 1522 | MF: ATP binding; BP: peptidyl-tyrosine phosphorylation; MF: non-membrane spanning protein tyrosine kinase activity |
| gi\|557771126\|ref\|XP_005185225\| | 5.09 | Proclotting enzyme | Serine proteinase stubble; [*D. navojoa*] | 63 | XP_017967821.1 | 0.0 | 817 | BP: proteolysis; MF: serine-type endopeptidase activity |
| gi\|557772014\|ref\|XP_005185665\| | 5.04 | Maltase A1-like | Maltase A1; [*D. ficusphila*] | 70 | XP_017044412.1 | 0.0 | 832 | BP: carbohydrate metabolic process; MF: catalytic activity; MF: cation binding |
| gi\|755851764\|ref\|XP_005175670\| | 5.04 | Tryptophan-tRNA ligase, cytoplasmic-like | Tryptophan--tRNA ligase, cytoplasmic; [*D. arizonae*] | 77 | XP_017856386.1 | 0.0 | 659 | MF: ATP binding; CC: cytoplasm; BP: tryptophanyl-tRNA aminoacylation; BP: tryptophan metabolic process; MF: tryptophan-tRNA ligase activity; BP: dendrite morphogenesis |
| gi\|557752368\|ref\|XP_005175934\| | 5.00 | Peroxiredoxin-2-like | Peroxiredoxin-2; [*D. suzukii*] | 78 | XP_016939860.1 | 3e-138 | 390 | BP: response to oxidative stress; BP: determination of adult lifespan; BP: negative regulation of apoptotic process; BP: obsolete peroxidase reaction; CC: mitochondrion; MF: thioredoxin peroxidase activity; BP: phagocytosis; BP: cellular oxidant detoxification; BP: cell redox homeostasis; BP: oxidation-reduction process; BP: hydrogen peroxide catabolic process |
| gi\|755887005\|ref\|XP_011294272\| | 4.98 | Hexaprenyldihydroxybenzoate methyltransferase, mitochondrial-like isoform X3 | Ubiquinone biosynthesis O-methyltransferase; [*D. miranda*] | 55 | XP_017153000.1 | 3e-103 | 306 | BP: metabolic process; MF: methyltransferase activity |
| gi\|557755250\|ref\|XP_005177366\| | 4.90 | Probable multidrug resistance-associated protein lethal(2)03659 | Multidrug resistance-associated protein 4; [*D. kikkawai*] | 50 | XP_017020280.1 | 0.0 | 1594 | MF: nucleotide binding; BP: drug transmembrane transport; MF: xenobiotic-transporting ATPase activity; CC: ATP-binding cassette (ABC) transporter complex |
| gi\|557785303\|ref\|XP_005192252\| | 4.89 | Uncharacterized protein LOC10189904 | Putative gag-pol protein; [*D. ananassae*] | 34 | ABP48078.1 | 0.0 | 684 |  |
| gi\|755866057\|ref\|XP_011291208\| | 4.85 | Uncharacterized protein LOC101887731 | Mucin-5AC; [*D. suzukii*] | 51 | XP_016937351.1 | 8e-124 | 423 |  |
| gi\|755874922\|ref\|XP_005183473\|***** | 4.73 | Uncharacterized protein LOC101895777 | CHKov1; [*C. quinquefasciatus*] | 35 | XP_001858906.1 | 8e-147 | 462 | BP: metabolic process; MF: transferase activity |
| gi\|755849177\|ref\|XP_011290729\|***** | 4.66 | Uncharacterized protein LOC101893836 | No Significant Hit found |  |  |  |  | CC: integral component of membrane |
| gi\|755899725\|ref\|XP_005191014\| | 4.63 | Hsp70 | Major heat shock 70 kDa protein Ba-like; [*D. suzukii*] | 86 | XP_016926536.1 | 0.0 | 1137 | MF: ATP binding |
| gi\|557775242\|ref\|XP_005187267\|***** | 4.59 | Uncharacterized protein LOC101900863 | Laminin subunit gamma-1; [*B. dorsalis*] | 47 | XP_011208045.1 | 4e-33 | 121 |  |
| gi\|557756834\|ref\|XP_005178152\| | 4.53 | Chymotrypsinogen 2 | Chymotrypsinogen A; [*D. kikkawai*] | 71 | XP_017037451.1 | 0.0 | 528 | BP: proteolysis; MF: serine-type endopeptidase activity |
| gi\|755878006\|ref\|XP_011292936\|***** | 4.51 | Uncharacterized protein LOC105261867 | No Significant Hit found |  |  |  |  |  |
| gi\|557757014\|ref\|XP_005178240\| | 4.50 | Calpain-C | Calpain-C; [*D. navojoa*] | 73 | XP_017964491.1 | 0.0 | 1056 | MF: calcium ion binding; BP: proteolysis; MF: calcium-dependent cysteine-type endopeptidase activity; CC: intracellular |
| gi\|557772014\|ref\|XP_005185665\| | 4.48 | Maltase A1-like | Maltase A1; [*D. ficusphila*] | 70 | XP_017044412.1 | 0.0 | 832 | BP: carbohydrate metabolic process; MF: catalytic activity; MF: cation binding |
| gi\|557776412\|ref\|XP_005187846\| | 4.47 | Cuticle protein 8 | Cuticle protein 8; [*D. ficusphila*] | 81 | XP_017042662.1 | 5e-71 | 213 | BP: chitin-based cuticle development; MF: structural constituent of chitin-based larval cuticle; CC: extracellular matrix |
| gi\|755892420\|ref\|XP_011295063\| | 4.43 | Acyl-protein thioesterase 1-like | Acyl-protein thioesterase 1; [*D. takahashii*] | 83 | XP_016992466.1 | 1e-135 | 383 | BP: RNA phosphodiester bond hydrolysis, endonucleolytic; MF: ribonuclease P activity; BP: regulation of RNA metabolic process; BP: tRNA 5'-leader removal; CC: ribonuclease P complex |
| gi\|755894298\|ref\|XP_011295337\| | 4.40 | Uncharacterized protein LOC105262331 | Blastopia polyprotein; [*D. melanogaster*] | 42 | CAA81643.1 | 1e-64 | 226 | BP: macromolecule metabolic process; MF: binding; BP: primary metabolic process |
| gi\|755882617\|ref\|XP_011293643\| | 4.33 | Protein tincar | Tinc; [*D. busckii*] | 54 | ALC46505.1 | 0.0 | 938 | BP: regulation of eye photoreceptor cell development; CC: plasma membrane |
| gi\|557782049\|ref\|XP_005190640\| | 4.31 | Protein dachsous-like | Protein dachsous; [*D. takahashii*] | 60 | XP_017011514.1 | 0.0 | 1717 | BP: cell proliferation; BP: imaginal disc-derived leg morphogenesis; BP: calcium-dependent cell-cell adhesion via plasma membrane cell adhesion molecules; BP: wing disc pattern formation; BP: ommatidial rotation; BP: cell morphogenesis involved in differentiation; BP: signal transduction; BP: heterophilic cell-cell adhesion via plasma membrane cell adhesion molecules; BP: regulation of establishment of planar polarity; BP: microtubule cytoskeleton organization involved in establishment of planar polarity; BP: homophilic cell adhesion via plasma membrane adhesion molecules; MF: receptor activity; BP: equator specification; MF: signal transducer activity; CC: integral component of plasma membrane; MF: calcium ion binding; MF: cadherin binding; BP: cell-cell adhesion mediated by cadherin; BP: establishment of epithelial cell apical/basal polarity; BP: establishment of imaginal disc-derived wing hair orientation; BP: peptide cross-linking; BP: regulation of tube length, open tracheal system |
| gi\|557778339\|ref\|XP_005188800\| | 4.30 | Neuropeptide-like 3 | Pupal cuticle protein G1A-like; [*D. ficusphila*] | 61 | XP_001957191.1 | 4e-30 | 107 |  |
| gi\|557774246\|ref\|XP_005186773\| | 4.29 | DEP domain-containing protein DDB_G0279099 | Probable serine/threonine-protein kinase DDB_G0267686; [*D. arizonae*] | 56 | XP_017858615.1 | 5e-128 | 383 | MF: DNA binding |
| gi\|755857759\|ref\|XP_005177649\| | 4.28 | Laccase-2-like | Laccase-2; [*D. arizonae*] | 91 | XP_017865494.1 | 0.0 | 1456 | BP: chitin-based cuticle development; MF: copper ion binding; BP: oxidation-reduction process; MF: hydroquinone:oxygen oxidoreductase activity |
| gi\|557750369\|ref\|XP_005174948\| | 4.26 | Larval cuticle protein 8-like isoform X1 | Larval cuticle protein 8-like; [*D. suzukii*] | 84 | XP_016934999.1 | 9e-51 | 159 | MF: structural constituent of cuticle |
| gi\|557761259\|ref\|XP_005180348\|***** | 4.24 | uncharacterized protein LOC101894483 | No Significant Hit found |  |  |  |  | CC: membrane; CC: integral component of membrane |
| gi\|755888617\|ref\|XP_011294503\| | 4.23 | Fibrinogen alpha chain | Angiopoietin-related protein 3; [*D. arizonae*] | 81 | XP_017868643.1 | 0.0 | 565 |  |

**Table S7. Comparative transcriptome analysis.** Comparison of the frequency of RNA-Seq reads of the MdSGHV infected libraries (V1, V2, and V3) mapped to all the 108 ORFs in the viral genome to the previously 454 reads generated from cDNA generated from a total RNA extracted from a pool of flies sampled at 2, 4 and 6 d-pi.

| **MdSGHV ORF description** | | | **MdSGHV-treatments** | | | **RNA-Seq reads frequency** | |
| --- | --- | --- | --- | --- | --- | --- | --- |
| **ORF name (Description)** | **S or NS (peptide) ²** | **Length [aa]** | **V1** | **V2** | **V3** | **Average reads ³** | **454 reads ^4^** |
| MdSGHV037 (SNAP190-like) | NS | 885 | 365722 | 364146 | 140580 | 290,149 | 1318 |
| MdSGHV086 | S (171) | 1186 | 354654 | 331163 | 143632 | 276,483 | 1121 |
| MdSGHV096 | S (478) | 4422 | 334500 | 339837 | 154074 | 276,137 | 384 |
| MdSGHV048 (RESA) | NS | 1173 | 199514 | 258453 | 99732 | 185,900 | 818 |
| MdSGHV040 | S (161) | 1827 | 203198 | 201279 | 79569 | 161,349 | 440 |
| MdSGHV093 | NS | 852 | 210160 | 177249 | 84755 | 157,388 | 812 |
| MdSGHV082 (RCI-recombinase) | NS | 1080 | 174893 | 188008 | 57892 | 140,264 | 394 |
| MdSGHV071 | S (7) | 1002 | 130301 | 125527 | 59690 | 105,173 | 382 |
| MdSGHV047 (ODV-E66) | S (121) | 2103 | 98508 | 90201 | 38717 | 75,809 | 44 |
| MdSGHV085 | S (5) | 1212 | 98364 | 85156 | 41385 | 74,968 | 365 |
| MdSGHV023 | S (56) | 771 | 92619 | 82274 | 33647 | 69,513 | 423 |
| MdSGHV028 | S (17) | 1146 | 80895 | 68098 | 42353 | 63,782 | 23 |
| MdSGHV097 | S (39) | 1449 | 78484 | 64472 | 33666 | 58,874 | 22 |
| MdSGHV092 | NS | 222 | 62436 | 67973 | 22178 | 50,862 | 745 |
| MdSGHV016 | S (3) | 1500 | 70473 | 50678 | 28873 | 50,008 | 35 |
| MdSGHV026 | NS | 183 | 58191 | 61426 | 22760 | 47,459 | 586 |
| MdSGHV055 | NS | 1251 | 54479 | 55864 | 24187 | 44,843 | 35 |
| MdSGHV022 | S (66) | 1032 | 54828 | 48814 | 20300 | 41,314 | 17 |
| MdSGHV033 (AAA+_ATPase) | S (68) | 1494 | 47086 | 42566 | 21965 | 37,206 | 10 |
| MdSGHV049 (Ac150) | NS | 504 | 37074 | 50677 | 20678 | 36,143 | 125 |
| MdSGHV020 | S (4) | 315 | 35142 | 39037 | 16534 | 30,238 | 559 |
| MdSGHV003 | NS | 471 | 39538 | 31243 | 18112 | 29,631 | 12 |
| MdSGHV025 | S (7) | 1131 | 35459 | 34434 | 15920 | 28,604 | 285 |
| MdSGHV064 (Iduna) | NS | 1428 | 40644 | 23652 | 21291 | 28,529 | 5 |
| MdSGHV032 | S (3) | 1182 | 36137 | 28915 | 14876 | 26,643 | 20 |
| MdSGHV094 (MKS3) | S (27) | 336 | 32592 | 31928 | 12999 | 25,840 | 91 |
| MdSGHV077 | NS | 897 | 30009 | 31664 | 11934 | 24,536 | 3 |
| MdSGHV029 (PIF-1) | NS | 1935 | 27216 | 21425 | 13559 | 20,733 | 6 |
| MdSGHV073 | NS | 1173 | 27583 | 20176 | 12551 | 20,103 | 2 |
| MdSGHV030 | NS | 2079 | 25933 | 17526 | 15225 | 19,561 | 10 |
| MdSGHV084 | NS | 1530 | 25217 | 18819 | 12052 | 18,696 | 2 |
| MdSGHV013 | S (6) | 1935 | 23249 | 19757 | 11279 | 18,095 | 5 |
| MdSGHV010 (MC) | NS | 771 | 19783 | 21718 | 10374 | 17,292 | 11 |
| MdSGHV090 | S (10) | 2019 | 23308 | 17721 | 9967 | 16,999 | 17 |
| MdSGHV076 | NS | 1149 | 23085 | 16747 | 8359 | 16,064 | 4 |
| MdSGHV072 (MPPE) | S (9) | 411 | 19562 | 18926 | 7668 | 15,385 | 251 |
| MdSGHV035 | NS | 729 | 20382 | 16653 | 8284 | 15,106 | 29 |
| MdSGHV099 | NS | 1839 | 18026 | 15039 | 9482 | 14,182 | 10 |
| MdSGHV083 | NS | 5343 | 19140 | 13742 | 9437 | 14,106 | 12 |
| MdSGHV043 | NS | 1110 | 16361 | 13441 | 12391 | 14,064 | 6 |
| MdSGHV062 (RR2) | NS | 1032 | 16112 | 13592 | 10056 | 13,253 | 22 |
| MdSGHV002 | NS | 582 | 17770 | 13629 | 7800 | 13,066 | 49 |
| MdSGHV068 | NS | 399 | 14961 | 16314 | 7377 | 12,884 | 36 |
| MdSGHV066 (CNT2) | NS | 1740 | 13910 | 11361 | 12642 | 12,638 | 13 |
| MdSGHV021 | NS | 1935 | 15287 | 13285 | 7100 | 11,891 | 10 |
| MdSGHV015 | NS | 900 | 14312 | 12656 | 8086 | 11,685 | 21 |
| MdSGHV100 | NS | 1638 | 14025 | 12571 | 7886 | 11,494 | 19 |
| MdSGHV044 | S (17) | 936 | 14309 | 14040 | 5662 | 11,337 | 4 |
| MdSGHV004 | NS | 396 | 15281 | 11679 | 6441 | 11,134 | 12 |
| MdSGHV070 | NS | 2904 | 14160 | 10133 | 8637 | 10,977 | 0 |
| MdSGHV038 | S (20) | 753 | 14184 | 12893 | 5664 | 10,914 | 147 |
| MdSGHV050 (ACY1) | S (4) | 1215 | 14992 | 12096 | 5430 | 10,839 | 5 |
| MdSGHV091 | S (2) | 1272 | 14059 | 9562 | 6872 | 10,164 | 6 |
| MdSGHV046 (LCAT) | NS | 1188 | 13850 | 10194 | 6075 | 10,040 | 6 |
| MdSGHV081 | NS | 285 | 14042 | 9692 | 5929 | 9,888 | 4 |
| MdSGHV056 | NS | 256 | 12075 | 12084 | 5082 | 9,747 | 1 |
| MdSGHV102 | S (6) | 774 | 12109 | 10675 | 6382 | 9,722 | 36 |
| MdSGHV057 | NS | 222 | 9902 | 11642 | 5642 | 9,062 | 9 |
| MdSGHV001 (DNA pol) | NS | 2934 | 10985 | 9031 | 6561 | 8,859 | 11 |
| MdSGHV107 | NS | 3338 | 11898 | 7871 | 6793 | 8,854 | 3 |
| MdSGHV101 | NS | 597 | 8413 | 9489 | 5639 | 7,847 | 85 |
| MdSGHV065 (RR1) | NS | 2358 | 9830 | 6902 | 6726 | 7,819 | 5 |
| MdSGHV014 (mopB) | S (20) | 750 | 9260 | 9396 | 4276 | 7,644 | 8 |
| MdSGHV088 | NS | 540 | 10571 | 7256 | 4957 | 7,595 | 12 |
| MdSGHV080 ^1^ | NS | 285 | 10149 | 6770 | 4093 | 7,004 | 3 |
| MdSGHV027 | NS | 510 | 8195 | 7965 | 4794 | 6,985 | 2 |
| MdSGHV011 (DHFR) | NS | 513 | 8002 | 8786 | 3484 | 6,757 | 2 |
| MdSGHV031 | NS | 201 | 9362 | 7413 | 3459 | 6,745 | 121 |
| MdSGHV052 (dUTP | S (3) | 453 | 8155 | 7128 | 4604 | 6,629 | 9 |
| MdSGHV074 | NS | 2097 | 8899 | 6085 | 3924 | 6,303 | 4 |
| MdSGHV042 | NS | 1884 | 7704 | 6093 | 4889 | 6,229 | 4 |
| MdSGHV045 | NS | 1143 | 8427 | 6302 | 3454 | 6,061 | 6 |
| MdSGHV039 ^1^ (P74) | S (8) | 2124 | 8438 | 5888 | 3616 | 5,981 | 13 |
| MdSGHV089 (PIF-2) | NS | 1140 | 8198 | 5664 | 3955 | 5,939 | 4 |
| MdSGHV012 (TS) | NS | 885 | 5088 | 6519 | 4657 | 5,421 | 7 |
| MdSGHV017 | NS | 1662 | 7022 | 5368 | 3299 | 5,,230 | 6 |
| MdSGHV079 | NS | 369 | 6239 | 6735 | 2534 | 5,169 | 14 |
| MdSGHV053 | NS | 441 | 6976 | 5926 | 2444 | 5,115 | 9 |
| MdSGHV069 (TK) | NS | 612 | 5609 | 5838 | 3886 | 5,111 | 9 |
| MdSGHV041 ^1^ | NS | 1326 | 6325 | 4598 | 4146 | 5,023 | 3 |
| MdSGHV036 (MMP-14) | NS | 591 | 6981 | 4925 | 2864 | 4,923 | 147 |
| MdSGHV087 | NS | 1635 | 6145 | 4520 | 3958 | 4,874 | 6 |
| MdSGHV108 (Ac81) | NS | 618 | 6035 | 5002 | 2777 | 4,605 | 8 |
| MdSGHV095 | NS | 423 | 4788 | 5309 | 3069 | 4,389 | 10 |
| MdSGHV104 (DNA helicase) | NS | 2313 | 4549 | 3174 | 2316 | 3,346 | 1 |
| MdSGHV054 | NS | 486 | 4371 | 4026 | 1531 | 3,309 | 11 |
| MdSGHV105 | NS | 729 | 4228 | 3356 | 2343 | 3,309 | 4 |
| MdSGHV067 | NS | 489 | 3792 | 3531 | 2451 | 3,258 | 6 |
| MdSGHV075 | NS | 327 | 3702 | 2600 | 1341 | 2,548 | 14 |
| MdSGHV098 | NS | 1215 | 2990 | 2245 | 1925 | 2,387 | 2 |
| MdSGHV051 | NS | 453 | 2886 | 1900 | 2017 | 2,268 | 4 |
| MdSGHV019 ^1^ | NS | 678 | 2556 | 1869 | 1113 | 1,846 | 6 |
| MdSGHV034 | NS | 195 | 2167 | 1897 | 1360 | 1,808 | 5 |
| MdSGHV018 | NS | 702 | 1943 | 2246 | 1176 | 1,788 | 35 |
| MdSGHV024 | NS | 1131 | 1766 | 1424 | 911 | 1,367 | 7 |
| MdSGHV058 | NS | 396 | 1651 | 1455 | 629 | 1,245 | 1 |
| MdSGHV106 ^1^ (PIF-3) | NS | 729 | 1270 | 834 | 644 | 916 | 0 |
| MdSGHV078 (IAP) | NS | 429 | 1238 | 1020 | 460 | 906 | 2 |
| MdSGHV063 | NS | 273 | 833 | 578 | 270 | 560 | 6 |
| MdSGHV061 (Transposase) | NS | 324 | 486 | 461 | 149 | 365 | 3 |
| MdSGHV103 | NS | 204 | 219 | 252 | 147 | 206 | 1 |
| MdSGHV059 ^1^ | NS | 198 | 161 | 185 | 46 | 131 | 0 |
| MdSGHV005 | NS | 210 | 50 | 47 | 51 | 49 | 0 |
| MdSGHV060 | NS | 156 | 50 | 60 | 30 | 47 | 0 |
| MdSGHV006 | NS | 195 | 34 | 50 | 32 | 39 | 0 |
| MdSGHV009 | NS | 255 | 39 | 16 | 35 | 30 | 0 |
| MdSGHV008 | NS | 252 | 30 | 23 | 15 | 23 | 0 |
| MdSGHV007 ^1^ | NS | 162 | 3 | 6 | 3 | 4 | 1 |

^1^Shaded cells in first column represent ORFs that were undetected (by 3' RACE and qPCR) by Salem *et al*., 2009 (see manuscript text for details).

^2^Peptide numbers taken from Garcia-Maruniak *et al*., 2008 and represent a compilation of LC-MS/MS data of purified enveloped MdSGHV.

^3^Colors in Average reads column depict highly abundant (red more than 5.0 x 10^4^ reads/library), abundant (yellow 1- 5 x10^4^ reads/library), moderate blue had between 2- 10 x10^3^ reads/library, low (green 2- 20 x10^2^ reads /library) and rust rare <100 reads (Figs.1, 2S).

^4^The 454 sequences were compiled from

1. **†** Deceased [↑](#footnote-ref-1)
